# Epidemiology and economics of foot-and-mouth disease: current understanding and knowledge gaps

**DOI:** 10.1186/s13567-025-01561-5

**Published:** 2025-07-07

**Authors:** John M. Humphreys, Carolina Stenfeldt, Donald P. King, Theodore Knight-Jones, Andres M. Perez, Kimberly VanderWaal, Michael W. Sanderson, Antonello Di Nardo, Wudu T. Jemberu, Nakarin Pamornchainavakul, Jonathan Arzt

**Affiliations:** 1 Agricultural Research Service, National Bio and Agro-Defense Facility, U.S. Department of Agriculture, Manhattan, KS USA; 2 Plum Island Animal Disease Center, Agricultural Research Service, U.S. Department of Agriculture, Greenport, NY USA; 3 Department of Diagnostic Medicine/Pathobiology, Kansas State University, Manhattan, KS USA; 4 The Pirbright Institute, Woking Surrey, UK; 5 International Livestock Research Institute, Addis Ababa, Ethiopia; 6 Veterinary Population Medicine, University of Minnesota, St. Paul, MN USA; 7 Center for Outcomes Research and Epidemiology, Kansas State University, Manhattan, KS USA

**Keywords:** Foot-and-mouth disease, FMD, epidemiology, global trends, pathogenesis, molecular, wildlife, modeling, economics

## Abstract

Foot-and-mouth disease virus (FMDV) is one of the few veterinary pathogens that defines policy and global trade in animal products. Its prominence necessitates approaches to control that integrate the multiple factors contributing to the disease’s biology and transmission characteristics. Central to this concept is the epidemiological FMD status (endemic or FMD-free, with or without vaccination) of a territory, which defines access to export markets. FMD epidemiology is complex, insufficiently understood, and intertwined with the biology of the virus (multiple serotypes and subtypes), global distribution (distinct regional virus pools), pathogenesis (subclinical infections and species differences), and host range (broad range of susceptible domestic and wild animals). Despite steady advances, critical knowledge gaps persist in FMD epidemiology that undermine the optimal control of FMD. This review summarizes the distinct thematic compartments of FMD epidemiology and presents the critical knowledge gaps that continue to limit the effectiveness of global, regional, and national initiatives to control and eradicate FMD.

## Introduction

Foot-and-mouth disease (FMD) is a multispecies viral disease that affects cloven-hooved livestock, including cattle, sheep, goats, pigs, and water buffalo [1]. Beyond these domesticated animals, FMD can infect a range of wildlife species, with African buffalo (*Syncerus caffer*) serving as the primary reservoir for the Southern Africa Territories (SAT) serotypes in sub-Saharan Africa [2]. The disease remains one of the most significant transboundary animal health challenges, restricting trade in animals and animal-derived products and leading to substantial socioeconomic disruptions. The complex epidemiology of FMD, driven by diverse hosts, rapid virus evolution, high transmissibility, and subclinical maintenance, shapes international trade policies and presents continued obstacles to effective control and eradication efforts.

Recognizing these challenges and the need for coordinated solutions, the Global Foot-and-Mouth Disease Research Alliance (GFRA) convened an FMD Gap Analysis Workshop in Buenos Aires, Argentina, on December 5–6, 2022. This cohort of international FMD researchers reviewed recent advances, identified critical knowledge gaps, and set priorities for future work to enhance global control and eradication strategies. The workshop defined distinct yet interrelated compartments of FMD research, including virology, pathogenesis, vaccinology, epidemiology, and immunology. Within FMD epidemiology, key domains of progress and associated knowledge gaps included global trends, molecular epidemiology, disease modeling, pathoepidemiology, wildlife interactions, and economic considerations. Each domain contributes complementary insights that, when integrated, inform FMD understanding, prioritize research needs, and highlight opportunities for interdisciplinary collaboration.

This paper synthesizes the workshop’s findings, presenting a comprehensive review of FMD epidemiology and an integrated gap analysis across the focal areas. Beginning with global trends in FMD epidemiology, the paper then considers how pathogenesis affects epidemiology, detailing the implications of subclinical infections, transmission pathways, and host-specific susceptibilities. Subsequent sections address advances and gaps in molecular epidemiology, emphasizing the need for robust genomic studies and analytical methodologies to track viral evolution and transboundary movements. Recognizing that wildlife hosts serve as reservoirs and bridges for species, the next section examines the wildlife-livestock interface and the ecological drivers of FMD spread and persistence across landscapes. The role of computational and mathematical modeling in predicting outbreak dynamics and optimizing control strategies is critically examined, with particular attention to parameterization challenges and contextual differences between FMD-endemic and FMD-free regions. Finally, the economic impacts of FMD and the cost-effectiveness of current and potential control strategies are explored, underscoring the necessity for standardized economic models that integrate epidemiological insights. 

By integrating evidence from multiple domains, this interdisciplinary review aims to guide future research and inform policy development, addressing immediate and long-term challenges in FMD control. It identifies critical gaps and priority topics for investigation, as well as opportunities for synergistic, cross-disciplinary collaboration to enhance our collective capacity to prevent and mitigate the global impacts of FMD.

## Global trends

### Regional endemic pools and transboundary spread

**Figure 1:**
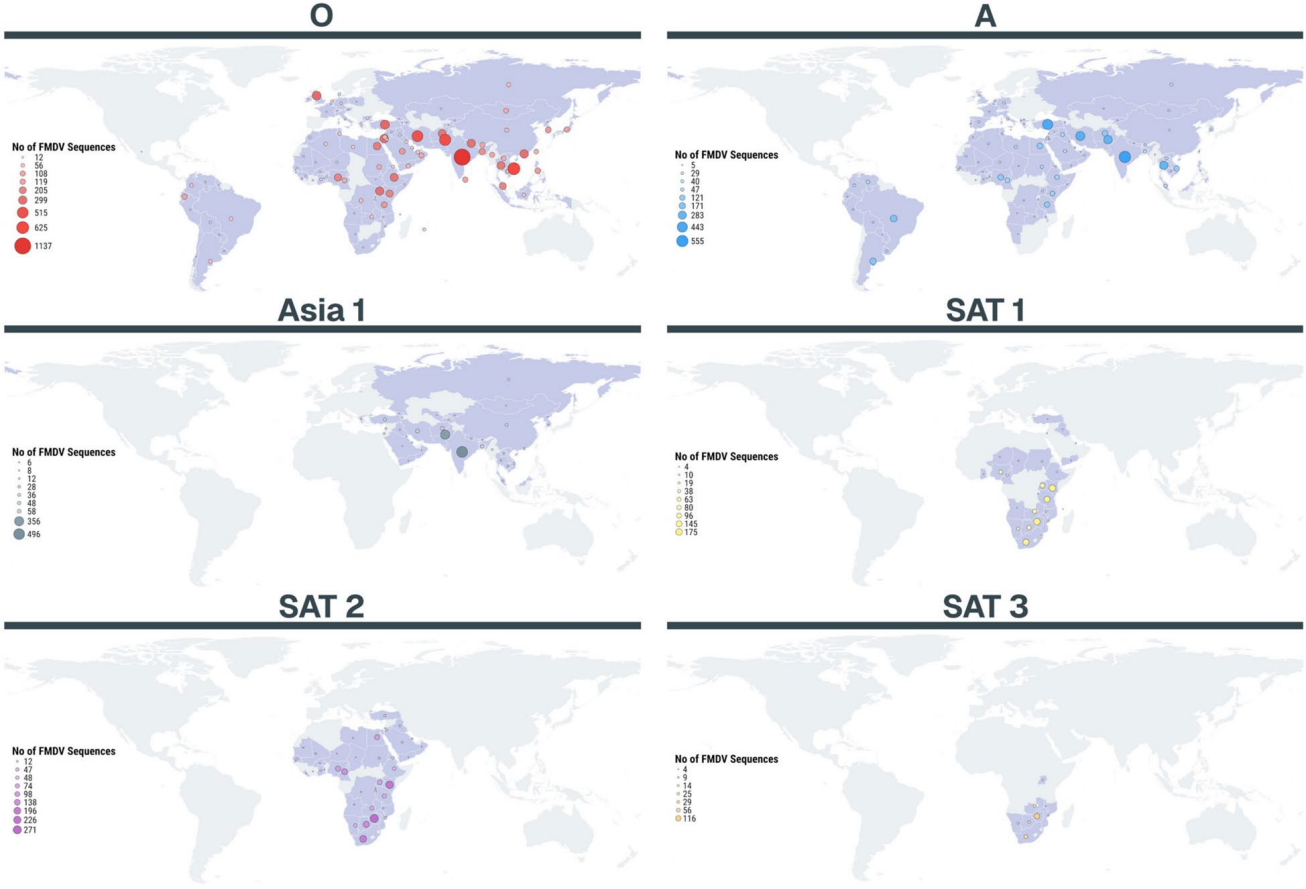
**Geographical distribution of FMDV sequence data that are available for the six circulating serotypes**. Data shown represent sequences available at www.FMDbase.org. NB: data for serotype C are not shown since this serotype has not been detected anywhere globally since 2004.

Global movements of foot-and-mouth disease virus (FMDV, family: *Picornaviridae*, species: *Aphthovirus vesiculae*) over the last decade have continued to reflect the historic trends that the virus is typically transported within infected animals and through anthropogenic actions, for example, fomites, illegal import/export, and the feeding of contaminated food products. FMD exists in seven distinct endemic pools [3] in areas of Asia, Africa, and South America, each pool maintaining a spectrum of different viral lineages within the six circulating serotypes (O, A, Asia 1, SAT 1–3; Figure 1). The seventh serotype, serotype C, has not been reported anywhere globally since the last field cases occurred in 2004 and appears now to be extinct [4]. The pool concept is a useful model that helps to understand the risks posed by specific FMD viruses, known as topotypes, which represent genetically and geographically distinct variants that circulate and evolve within endemic regions. The geographical borders of these seven pools are not strictly defined and are thought to be determined by the prevailing trade patterns in livestock and livestock products that underpin regional source-sink metapopulation dynamics. Viral sequence data can be used to document cycles of infection originating from different endemic lineages [5] as well as to reconstruct the common virus transmission pathways (viral conveyors) within these pools [6]. Particular attention is often given to trans-pool movements because introducing new FMDV lineages can dramatically impact a region’s risk profile and the suitability of vaccines for controlling outbreaks, especially if there is no pre-existing immunity from previous infection or vaccination.

Since 2015, global FMD epidemiology has been dominated by the spread of Ind-2001 lineages of serotype O (O/ME-SA/Ind-2001a-e lineages) from South Asian countries (Pool 2) to cause outbreaks in the Gulf States of the Middle East and Pakistan (Pool 3) [7, 8]. This lineage has also spread to Southeast Asia (Pool 1, [9]), where it is now more frequently reported than the other serotype O lineages that were previously dominant in the region, such as O/SEA/Mya-98 and O/ME-SA/PanAsia. The upsurge of FMD cases in Southeast Asia has preempted the onward spread of viruses beyond Pool 1 to cause outbreaks in new locations such as in South Korea [10], Russia and Mongolia [11], and countries and zones that were previously FMD-free without vaccination, including Indonesia [12] and Kazakhstan [13].

Over the past two years, events in the European vicinity have been dominated by the emergence of the SAT2/XIV topotype in Western Asia, with reports in Iraq, Jordan, Türkiye, Bahrain, and Oman. Phylogenetic analysis demonstrated that multiple introductions of SAT2/XIV viruses originating from East Africa have occurred in the affected countries [14]. The unexpected detection of the SAT1/I topotype in Qatar (during 2023) further demonstrates the current epidemiological connections between East Africa and the Middle East via livestock trade routes. In North Africa, outbreaks in Algeria, detected in December 2023, represent the first reports of the SAT 2 serotype in the Maghreb. The emergence of SAT 2 in the region is not entirely unexpected, given that the SAT2/VII topotype widely circulates in West Africa [15] and has been identified as a risk for spreading into North Africa following similar pathways to serotypes O and A [16, 17]. However, identifying SAT2/V as the causative virus was surprising because this topotype had not been detected anywhere since 1991. Furthermore, there have been reports of FMD cases in Egypt (2022) associated with viruses from the O/EURO-SA [18] and A/EURO-SA [19] topotypes that are usually restricted to South America.

For Southern Africa (Pool 6), detecting the O/EA-2 topotype represents the first time in 20 years that serotype O has been found in this region, where serotype O vaccines are not widely used [20]. South Africa has also lost its official WOAH FMD-free (without vaccination) status due to outbreaks of the SAT 1, SAT 2, and SAT 3 serotypes. Taken together, these events highlight the ease with which new FMDV lineages can emerge and cross international boundaries and the importance of the work to continuously monitor global FMD epidemiology.

### Surveillance challenges and underreporting

Global FMD surveillance remains fragmented and is often shaped by political willingness to report cases, as well as the availability of local veterinary capacity to detect outbreaks and collect diagnostic samples. It is well known that the clinical presentation of FMD varies across different susceptible species, as FMD is less apparent in small ruminants and certain local livestock breeds [21]. Furthermore, FMD vaccination may result in neoteric subclinical cases where animals are infectious but lack clinical signs, thereby making it more challenging to identify infections and trace transmission routes. Therefore, understanding FMD epidemiology requires assembling sparse and uneven data that are inherently biased and complicated by the unequal geographic distribution of FMDV serotypes and variation across different host species. Disclosure of information relating to FMD outbreaks plays a crucial role in a more detailed understanding of transboundary risk pathways for FMD. To this end, initiatives such as the WOAH/FAO FMD Reference Laboratory Network, which aim to build trust and promote open data sharing, are valuable in providing near real-time snapshots of strain migrations. Despite those efforts, sampling and testing only catch a minority of outbreaks, with most FMD clinical cases remaining undetected and unsampled.

It is widely recognized that in endemic regions, outbreaks represent only the “tip of the iceberg” of the true FMDV burden, with viruses predominantly maintained through distinct forms of subclinical infection, known as neoteric and persistent infections [22]. Neoteric infections refer to recently acquired subclinical infections that occur during the “acute phase” or early stages of viral replication and shedding. In contrast, persistent subclinical infections occur when animals lack signs but are FMDV carriers beyond the acute phase, harboring the virus for extended periods, thereby contributing to long-term viral maintenance and transmission. Thus, the passive surveillance process of sampling from clinically observable cases fails to capture the diversity in subclinically infected animals. Active surveillance continues to provide valuable insights into the emergence and subclinical circulation of FMDV strains in Asia and Africa; however, active surveillance is labor-intensive and impractical to apply consistently in all endemic regions. Furthermore, the contagion potential from subclinically infected animals remains controversial.

### Serological and molecular tools

Serological surveys can be adopted to estimate the prevalence of FMD in endemic regions. Antibody testing targeting non-structural proteins (NSPs) is the most commonly used approach, as these assays are pan-serotypic and widely available from numerous commercial providers. Studies have demonstrated that some NSP-specific ELISA kits yield comparable results and can be used interchangeably for testing field sera. These tests are beneficial for uncovering evidence of FMDV infection in locations where active surveillance is difficult or where transmission is suspected in small ruminants, from which clinical signs may be difficult to recognize [23]. The systematic design of these surveys plays a critical role in the qualitative value of the surveillance data. Where the history of animals is uncertain, sampling often focuses on young stock (< 6 months of age) because older animals may have NSP-specific antibodies arising from previous infections, as a by-product of unpurified vaccines, or as a result of having been administered multiple doses of purified vaccines. The specificity of these tests is < 100%, therefore, false positives can be expected, and associated studies require that testing results be carefully analyzed to reveal spatial or temporal clustering. Although widely employed, these serological studies are often geographically limited, typically confined to targeted surveillance areas, and are rarely integrated or coordinated at a broader regional scale. A weakness of NSP testing is that it is not possible to disentangle the component antibody responses that arise from infection with more than one FMDV serotype. Structural protein (SP) ELISAs have high diagnostic sensitivity for FMDV-specific antibodies; however, their ability to accurately identify the correct FMDV serotype is limited, even when a monovalent serotype of known provenance is tested [24]. For these reasons, the virus neutralization test (VNT) remains a standard method for assessing immune responses following infection or vaccination, although it too has notable limitations.

The global circulation of different FMDV serotypes and lineages is characterized by a complex interplay between factors, such as viral evolution that generates antigenic novelty and the trade patterns that underpin the movement of animals. The apparent disappearance of serotype C raises important questions about the relative fitness of different FMD viruses across susceptible hosts, the regional connectivity between the seven endemic pools, and the population sizes required to maintain FMDV. These concerns have motivated work by the FAO/WOAH Serotype C Task Force to prevent the inadvertent re-emergence of this serotype, particularly through potential risks such as the use of improperly inactivated FMD vaccines. While population-level FMD dynamics represent the broadest scale of epidemiological strata, patterns at this level are underpinned by the micro-epidemiology of pathoepidemiology, including transmission biology and virus-host interactions. More specifically, the infectiousness and contagiousness of FMDV strains within distinct hosts determine fitness, emergence, and, ultimately, global relevance.

## Pathoepidemiology

### Pathogenesis and clinical syndrome

Pathogenesis in susceptible hosts plays a central role in shaping FMDV epidemiology. Factors such as the route of exposure, tissue-specific viral loads, and the magnitude and duration of viral shedding directly influence FMD transmission dynamics. These processes are further complicated by the virus’s broad host range, with species-specific differences giving rise to distinct patterns of infection and transmission. In particular, variation in the frequency and characteristics of subclinical and persistent infections among host species introduces critical epidemiological considerations. This intersection of pathogenesis and epidemiology is referred to as pathoepidemiology.

Classical clinical FMD is characterized by fever and vesicular lesions (blisters) in the mouth and on teats, coronary bands, and inter-digital clefts of the hoofs. The lesions are painful and cause varying degrees of hypersalivation and lameness, from which the disease gets its name [25, 26]. During early infection, animals are generally highly contagious due to large quantities of virus in secretions, blood, and tissues. In particular, vesicular epithelium and vesicular fluid have extremely high viral loads that serve as sources of transmission, either through direct contact, environmental or fomite contamination, or by resuspension into infectious aerosols.

Although the clinical condition can be debilitating, the severity of FMD varies across species. These contrasts are highlighted by FMDV circulation in the African buffalo (*Syncerus caffer*), which is known to be a wildlife reservoir for the SAT serotypes in Africa [27], but are typically unaffected by the clinical syndrome, even though the virus can spread rapidly within groups of naïve animals [28]. A similar phenomenon occurs in some indigenous cattle breeds in areas of endemic FMD, which are typically less affected by clinical FMD compared to imported breeds [29]. Clinical FMD in small ruminants is notoriously difficult to detect and recognize [30], even though these animals are highly susceptible to infection [31–35]. However, subclinical or mild clinical infection can be epidemiologically relevant, as was observed during the serotype O epidemic in Europe during 2001 [36].

### Subclinical FMD (neoteric and persistent infections)

The epidemiology of FMDV is complicated by two distinct forms of subclinical infection, which have important distinctions in pathogenesis and transmissibility. Early (acute) phase FMDV infection without clinical signs of disease is referred to as neoteric subclinical infection [22, 37]. Neoteric subclinical FMD typically involves the shedding of infectious virus in oronasal secretions, which makes the virus highly transmissible and differentiates neotericism from the FMDV carrier state. Neoteric infection may be due to pre-existing immunity from vaccination, previous exposures, or natural resistance to clinical FMD [37, 38]. Epidemiologically, the noteworthy point is that under appropriate neoteric conditions, a substantial spread of FMDV may occur, particularly through the movement of animals, without detection of clinical cases.

Persistent subclinical FMDV infection, commonly referred to as the FMDV carrier state, occurs at a high prevalence within ruminant hosts but not suids, following either clinical or subclinical primary infection, regardless of vaccination [38–41]. Various studies have updated the conventional wisdom that 50% of animals become carriers, demonstrating that > 85% of infected cattle may become carriers when modern detection techniques are applied [38, 41–44]. Depending on the host species, the FMDV carrier state involves low-level viral replication in the epithelial tissues of the upper respiratory (nasopharynx) or gastrointestinal (palatine tonsil) tract. In cattle, persistent FMDV has been localized to specific segments of lymphoid-associated epithelium of the nasopharyngeal mucosa [38, 42], whereas FMDV persistence in sheep has been demonstrated to occur in similar epithelium found within crypts of the oropharyngeal tonsils [32]. During persistent FMDV infection, the infectious virus can be recovered by scraping the mucosal surface with a specially designed probang cup [40, 45]. However, the virus is typically not present in oronasal- or other secretions [38, 46], and the contagion associated with FMDV carriers is therefore believed to be minimal. A handful of studies have failed to detect transmission of FMD from carriers [47–50]. However, a definitive determination of the plausibility of transmission from carriers remains elusive and controversial.

Recent experimental studies have demonstrated that recombination of FMDVs frequently occurs in the nasopharynx of carrier cattle that are super-infected with heterologous strains; thus, such animals are simultaneously carriers and neoterically infected [51]. It is, therefore, speculated that carrier cattle may contribute to FMDV diversity as these recombinant viruses are present in tissues and secretions from the upper respiratory tract during the neoteric subclinical phase of infection, where virus shedding may occur. However, this phenomenon has not yet been demonstrated under natural conditions.

### Transmission of FMDV

The efficiency of FMDV transmission varies across species and is, in part, determined by the exposure dose and route. The site of primary infection differs between species, and the risk associated with various exposure pathways depends on the specific tissues the virus must reach to initiate infection. Cattle and sheep are highly susceptible to aerogenous FMDV exposure [52, 53], consistent with their initial site of infection being localized to the nasopharynx, in the upper respiratory tract [32, 42, 54, 55]. By contrast, pigs are highly resistant to FMDV infection via inhalation but are more efficiently infected by the oral route [56]. Experimental studies have shown that the initial site of FMDV infection in pigs is localized to epithelial crypts of the oropharyngeal tonsils [57, 58], which is more consistent with ingestion of the virus or another oral exposure route. Although swill-feeding of pigs has been implicated as the initiating factor in major FMD epidemics, experimental studies have also shown that the viral dose required to cause FMD in pigs by feeding is surprisingly high [59]. Although pigs require a relatively high viral dose to become infected, this is offset by their ability to shed exceptionally large quantities of virus once infected [58], resulting in rapid transmission among group-housed individuals [60–63].

Interestingly, pigs efficiently clear FMDV from all tissues within approximately two weeks after infection, and there is no FMDV carrier state in pigs [39].

Because host susceptibility varies by species and route of exposure, it is essential to account for these differences during model parameterization to ensure an accurate representation of transmission risk. While ruminants such as cattle and sheep may become infected by breathing in the virus from some distance, pigs need to be in direct physical contact with each other for transmission to occur [64, 65]. By contrast, once pigs are infected, excessive quantities of FMDV can be isolated from their breath, and it is thus believed that pigs may function as a source of aerogenous FMDV transmission to other susceptible hosts [66–68].

Gaps exist in how aspects of pathogenesis inform FMDV epidemiology. Most FMD field surveys are focused on detecting and characterizing FMDV from clinical cases (outbreaks). It is clear from numerous sources that subclinical neoteric and persistent infections are common [37, 69–72] and that clinical FMD represents the “tip of the iceberg” of the FMDV present in endemic regions. However, there is a scant basis for quantifying or characterizing the risk of contagion associated with the movement of subclinically infected animals. As a result, this transmission compartment is generally excluded from modeling FMD outbreaks. Although the FMDV carrier state has been a known feature for multiple decades [40, 45], neoteric subclinical FMDV infections have received less attention. The occurrence of subclinical neoteric infections, during which infected animals may shed the virus without exhibiting clinical signs, increases the likelihood of unknowingly transporting infected animals and introducing the virus to new herds and regions before the disease is detected. Similarly, small ruminants are largely neglected in FMDV surveillance and modeling. Even though transmission from small ruminants is believed to be less efficient compared to other hosts [73, 74], it is clearly not a zero-risk condition, and excluding these animals from surveillance efforts creates a widespread deficit in understanding FMDV molecular epidemiology.

## Molecular epidemiology

FMDV’s rapid evolution, contagiousness, and diverse host range make it an ideal subject for molecular epidemiologic studies. Advances in molecular epidemiology have enhanced our understanding of the virus, including the interplay of susceptible hosts and environmental factors, which is essential for FMD control and eradication. Based on 92 studies from 2015 to 2024, this section of the review highlights four key perspectives: methodologies, genomic regions studied, spatial/temporal scales, and new findings, along with research gaps from the past decade.

More than half of the studies included in the analysis, averaging four to five published studies per year, focused on characterizing FMDV topotypes. Phylogenetic tree reconstruction and genetic comparisons with reference sequences have been used to identify and classify known and novel topotypes [23, 75–77]. These studies often compare the genetic relatedness of field viruses to local vaccine strains [78–81] and may conclude if an epidemic is linked to previously reported strains [82, 83], nearby circulating viruses [84, 85], or exotic topotypes [15, 86–88].

Many conclusions were drawn from phylogenetics, supplemented by serological tests and evolutionary insights, including amino acid profiles and analysis of selection pressure. Amino acid comparisons help capture protein mutations, while selection pressure analysis quantifies rates of site-specific changes through time [89, 90]. Most findings indicated that the P1 region is evolving under purifying selection, whereas specific surface antigenic sites are under diversifying selection [91–94], highlighting FMDV adaptation to infection dynamics and mitigation efforts like vaccination. Some studies examined variation in selection pressure across hosts, infection stages, temporally clustered sample sets, and individual animals [94–96].

Phylodynamic methods for quantifying viral spread are increasingly popular, with an average of two to three studies per year within the analyzed set. Sequences, sampling location, and date are key for inferring viral gene flow through phylogeography [97]. Large-scale analyses use countries or regions as discrete units to reveal transboundary spread [4, 7, 16, 98]. FMDV transmission can also be estimated between other traits, such as host species or clinical status [72, 95, 99–101], with trait-related data integrated as predictors in generalized linear models (GLMs) to assess their association with viral spread [6, 102, 103]. At a finer scale, the geographic coordinates of samples enable the estimation of viral dispersion velocity and link it to environmental factors [103–107], helping to identify risk factors for FMDV spread. Estimates of dispersal velocity of serotype O (approximately 10–71 km/month) in East Africa based on sequence-based phylogeographic approaches resulted in similar estimates as those produced by intensive fine-scale outbreak monitoring, demonstrating the utility of Bayesian phylogeographic approaches to infer viral dispersal patterns even in sparsely sampled endemic areas [5, 106].

Method development for transmission pathway inference between individual hosts, identifying “who infected whom”, frequently used FMDV outbreaks as a model host–pathogen system. Transmission tree models developed between 2015 and 2017 [108–111] were tested on the UK’s 2001 and 2007 FMDV outbreaks [112, 113], but practical use in recent outbreaks was limited [77, 94, 114, 115]. This gap between model development and application likely results from strict model assumptions that do not align with scenarios where host-level data is limited. However, as research on within- and between-host evolution and transmission dynamics grows, the use of transmission network analysis is expected to increase.

### Viral genomic regions used for analysis

In molecular epidemiology, the FMDV genetic sequence is the fundamental analytic input, and the genomic regions utilized vary across studies. Most studies (75%) use the VP1 coding region, with 85% of these relying solely on it, as it contains key antigenic domains for neutralization, aligning well with serotyping despite representing only 8% of the genome [116–120]. However, other regions are also important. VP2 and VP3 contribute to antigenicity [121], Lpro inhibits the antiviral responses [122], NSPs support replication and immune modulation [123], and the 5’ UTR’s S-fragment is essential for genome stability [124]. However, analysis of any individual segment may fail to capture the full evolutionary history of a virus since distinct genomic elements may have evolved separately and were then joined through recombination.

Using longer genomic segments like the P1 region or whole-genome sequences (WGS) in phylogenetic analyses offers broader insights but can lead to misinterpretations. Failure to account for recombination within regions that have different evolutionary histories may bias substitution rate estimates and phylogenetic accuracy [7, 125, 126]. A previous review found no significant difference in substitution rates between WGS and VP1, though rates varied [127]. Pedersen et al. underscored how sampling strategies affect inferred substitution rates [96], stressing caution when comparing rates across studies with differing sampling ranges.

To address recombination, a tree can be constructed from full-length sequences with recombinants removed or by splitting sequences into recombination-free fragments to build multiple trees [128]. The former approach is more suitable when there are few recombinant samples, while the latter provides higher accuracy [129]. Like other picornaviruses [130], analyses of large datasets reveal non-random recombination patterns in FMDV, with low recombination in structural genes (P1) but high levels in NSPs [51, 128, 131], possibly linked to genetic shifts via capsid switching. Thus, P1 or partial P1 [7] phylogenetic analysis may offer more precise evolutionary insights than VP1 alone, avoiding confounders from recombination present in other parts of the genome.

Studies on different genomic regions continue to refine the understanding of FMDV dynamics. Phylogenies of seven different genomic fragments from Africa revealed novel SAT genotypes, likely representing ancient viruses that were lost during the rinderpest pandemic [132]. Near-complete genome analysis in Southeast Asia linked two early endemic viral lineages despite differences in their capsid protein phylogeny [128], and a pandemic study highlighted phylogenetic inconsistencies between VP1 and WGS due to recombination [7]. These findings show the need for genomic studies beyond tracking transmission based on only VP1 variability [114].

Third-generation sequencing, like ONT’s MinION and PacBio’s SMRT, can support this with long-read capabilities, improved error rate, and rapid processing [133, 134].

However, Sanger and next-generation sequencing remain more widely used as long-read sequencing awaits established protocols in reference laboratories.

### Spatial and temporal scales of study

Molecular FMDV epidemiologic studies vary widely in scope. About half focus on local scales, targeting outbreaks in small regions within countries or border areas, with an average observation period of 1.3 years. Notably, 24% of local studies were in East Africa (Pool 4), mainly to characterize topotypes, followed by lesser proportions in West/Central Africa (Pool 5, 13%) and the Middle East (Pool 3, 13%). Frequent reports of FMDV emergence in East Africa likely stem from high levels of FMDV variability (five serotypes and over 40 topotypes), large livestock populations and trade, wild hosts, poor biosecurity, and porous borders [135, 136].

National-level studies comprised 30% of the total, with an average data collection period of 11 years. These were primarily from East Asia (Pool 1, 30%), South Asia (Pool 2, 30%), and East Africa (Pool 4, 26%). Over half included selection pressure analysis and phylogeography. These studies present a broader perspective on FMDV dynamics, for instance, the spread patterns and hotspots of O/PanAsia in Vietnam [95], distinct pathways of three serotypes in Ethiopia [105], and the drivers of serotype A evolution in Pakistan [137].

Regional-scale studies (16%) cover multiple countries within or across connected pools, using an average of 36 years of data, while global studies (3%) encompass multiple regions with data spanning 55 years. Half of the regional studies focused on a single pool, while the others investigated inter-pool interactions, addressing the long-distance or trans-pool spread of significant strains like the pandemic O/ME-SA/Ind-2001 [7] and the O/EA-3 in pools 4 and 5 [16]. Global studies explored FMDV’s long evolutionary history, including estimates of its ancient origins [131] and a complete tracing of the extinct serotype C [4].

### Novel findings from the past decade

Recent phylodynamic analyses have unveiled key FMDV transmission risk factors, such as cattle movements, high livestock density, and proximity to livestock markets in East Africa [103, 106, 107], while in Western and Southern Asia, shared borders and livestock trade are major drivers [6]. While critical for FMD control in these regions, these insights are not broadly applicable elsewhere, underscoring the need for comprehensive metadata collection and targeted research in other areas.

In East Africa, phylodynamic studies have yielded insights into region-specific drivers of FMDV transmission. Analyses incorporating viral genetic sequences with spatiotemporal metadata have shown that FMDV serotype O tends to persist and spread in areas characterized by high cattle density, proximity to livestock markets, and high human population density [106]. Further modeling efforts, utilizing step selection and resource gradient functions, identified low-rainfall regions as viral sinks that are frequently subject to reintroductions rather than sustained circulation. In contrast, high-density livestock regions near markets serve as consistent sources of viral dissemination [107]. These findings highlight the importance of both ecological and anthropogenic factors in influencing FMDV transmission in endemic systems.

The dominant role of livestock trade and market infrastructure in East Africa contrasts with patterns observed in Western and Southern Asia, where shared borders, transboundary livestock trade, and large, interconnected production systems are major drivers of virus spread [6, 103]. Recent phylodynamic analyses in this region have further demonstrated that Southern Asia functions as a key source region for viral emergence and westward dissemination, facilitated by livestock movements through ecological corridors and trade networks [6]. These regionally distinct dynamics underscore the importance of geographically tailored surveillance and control strategies and reinforce the need for context-specific risk assessments.

Challenges in FMD molecular epidemiologic studies include but are not limited to sampling and selection bias, phylogenetic confounding due to recombination, and unequal access to resources and data across regions. While the VP1 region remains the primary target for classification, other genomic areas are becoming increasingly valuable. The rise of WGS may soon necessitate standardized guidelines for other commonly used regions, such as P1 or Lpro. Although factors such as selection pressure and recombination have been documented, their impacts on persistence, transmission, and spread require further study. Advanced tools like genome-wide association studies (GWAS) and machine learning, combined with larger datasets, can potentially reveal FMDV’s key success factors. Although picornaviruses are known to naturally exist as a swarm of closely related variant viruses, molecular epidemiological analyses routinely treat viral isolates as individual static consensus sequences. An FMDV-infected animal is actually infected by a population of variants, containing sub-consensus sub-populations with distinct genomic and phenotypic properties [42]. Similarly, when FMDV transmits between animals or translocates across global pools, the virus moves as a swarm with the fitness defined at this subconsensus level. Affordable NGS has made subconsensus viral data readily available, yet including such viral characteristics in molecular epidemiological analyses is rarely achieved.

## Wildlife

### Wildlife-livestock interface dynamics

Wildlife populations influence FMD epidemiology in both endemic and non-endemic regions by acting as viral reservoirs, amplifiers, and transmission bridges that link domestic and wild hosts [2, 138, 139]. The interplay of ecology, animal behavior, and human activity at the wildlife-livestock interface shapes disease dynamics and underscores the need for comprehensive and integrative research approaches [140]. Such efforts can guide surveillance strategies, inform control strategies, and prioritize targeted research questions, ultimately contributing to more effective disease management [141–144]. A broad range of wildlife species have been identified as FMDV-susceptible under natural and experimental conditions [2]. Yet, the epidemiological relevance of these findings has been largely unexplored, with few exceptions.

Wildlife species can serve as long-term maintenance hosts for FMDV within endemic regions, particularly in sub-Saharan Africa. African buffalo (*Syncerus caffer*) remains the most thoroughly studied example, harboring SAT serotypes for extended periods and acting as primary wildlife reservoirs [2, 72, 145]. Buffalo can maintain infections in the absence of clinical signs, contaminating shared resources, such as watering holes and grazing areas [28, 146, 147] and creating persistent sources of infection for both wildlife and livestock [26, 143]. Interestingly, analysis of buffalo-collected FMDV sequences shows that fine-scale geographic features, such as rivers, appear to influence virus circulation and that social segregation among sympatric herds may limit between-herd transmission [136], highlighting the role of host behavioral ecology in determining epidemiological dynamics at the landscape scale. In addition to African buffalo, other wild ungulates, such as impalas.

(*Aepyceros melampus*) and kudus (*Tragelaphus strepsiceros*) play intermediary roles, bridging host communities and demonstrating the interconnectedness of FMD multi-host transmission networks [2]. Phylogeographic analyses linking wildlife infections to livestock outbreaks highlight the complexity of these systems, revealing regional variation driven by the distinctive agricultural practices and land-use patterns across Africa that shape wildlife–livestock interfaces [136]. This emphasizes the importance of expanded research efforts to characterize transmission pathways and the role of wildlife reservoirs more fully [72, 143]. Outside of endemic regions, Wildlife also complicates FMD control in FMD-free territories. Wild boars and feral domestic pigs, for instance, can accumulate FMDV loads comparable to those of domestic swine and shed FMDV before clinical signs become evident [138, 148–151]. The presence of Wild suids near livestock production zones, in combination with their capacity for undetected transmission, pose major surveillance challenges and increase the potential for rapid and undetected geographic spread following incursion events [138, 152]. Similar risks arise from various cervid species, including mule deer, white-tailed deer, roe deer, sika deer, and muntjac, which have demonstrated the capacity to sustain and spread FMDV. Several cervid species shed virus at levels similar to those of domestic livestock, suggesting that their ecological role in maintaining and dispersing FMDV may be more prominent than previously appreciated [143, 145, 153–155].

### Wildlife surveillance and diagnostic challenges

Detecting FMD in wildlife remains challenging because clinical presentations may differ substantially from those observed in livestock. Vesicular lesions, if present, may be subtle or atypical, hindering early recognition and enabling undetected viral spread [139, 149, 151]. Enhanced surveillance systems that utilize electronic tools, such as unmanned aircraft systems, camera traps coupled with machine-learning algorithms, and GPS tracking collars, could improve early detection and reveal cryptic infection hotspots [156]. Molecular and genetic technologies, including next-generation sequencing and environmental DNA (eDNA) sampling, provide sensitive approaches to help identify subclinical infections and guide targeted response measures [139, 157].

Environmental factors and human-mediated land use further shape wildlife-livestock disease dynamics. Habitat fragmentation, agricultural intensification, and diminished access to natural resources concentrate wild and domestic animals into shared foraging or watering points, creating “hotspots” of elevated contact and transmission risk [146, 147, 158]. In parts of South America, deforestation and altered land use patterns bring wildlife such as capybaras (*Hydrochoerus hydrochaeris*) into closer contact with cattle, increasing the likelihood of pathogen exchange [150].

Beyond direct animal-to-animal interactions, FMDV persistence in soils, organic matter, and wildlife carcasses has the potential to transform the landscape into an environmental reservoir [6, 22, 142, 159, 160]. Such non-direct transmission points complicate control efforts, as the inclusion of environmental decontamination and wildlife carcass disposal may be required as part of an effective operational response.

### Management, modeling, and policy

Livestock management practices likewise influence the type and frequency of wildlife-livestock interactions. Traditional pastoral systems in sub-Saharan Africa, characterized by communal grazing and routine movement of livestock, overlap extensively with wildlife habitats, thereby increasing contact opportunities [135, 136, 161]. Modern European operations rely on biosecurity measures; however, relatively smaller, peripheral farms near wildlife areas may serve as conduits or refugia between operations, creating vulnerable points in production landscapes [140, 162, 163]. Differences in management practices underscore the need to develop regionally tailored interventions that consider local ecological conditions, production methods, and prevailing socioeconomic realities. Epidemiological and ecological modeling remains a critical but underdeveloped tool for understanding FMD at the wildlife-livestock interface [154, 164]. Many current models struggle to incorporate wildlife-specific movements, ecological nuances, and seasonal resource availability, limiting their ability to accurately predict outbreak dynamics [72, 165–167]. Incorporating molecular and phylogeographic data with telemetry studies, remote sensing, and socioeconomic information could improve model parameterization and outbreak forecasting [147, 152]. For instance, high-resolution movement data from wildlife-tracking collars combined with satellite imagery of habitat changes may help identify potential corridors for virus transport, enabling more precise intervention targeting [164].

Management approaches to mitigate wildlife-related FMD risks must balance disease control, ecological integrity, and human livelihoods. Measures like fencing or grazing restrictions can limit direct interactions but may disrupt wildlife migrations, alter ecosystem function, and affect local livelihoods [146, 150]. Inspired by successful rabies control efforts, wildlife vaccination programs for FMDV may represent another potentially valuable strategy. However, practical challenges such as vaccine delivery, dosage, and species-specific immune responses, combined with the perceived relatively low contribution of wildlife to disease circulation in domesticated species, often diminish interest in vaccinating wildlife [28, 157]. Identifying effective and cost-efficient wildlife vaccination protocols that minimize environmental impacts and respect conservation goals remains an active research topic.

Current surveillance approaches, particularly those reliant on passive detection, often fail to identify wildlife infections before FMDV spills over into livestock [140, 143, 168]. Emerging technologies that move beyond passive detection, such as eDNA sampling, advanced wildlife tracking, and next-generation sequencing, can improve early detection and produce richer data on pathogen movements [138, 151, 156, 169, 170]. Adopting these tools could also facilitate more informed decisions about operational resource allocation, enabling targeted surveillance in high-risk areas.

Socioeconomic factors, local farming practices, and conservation policy profoundly affect wildlife-livestock interfaces [171]. East African pastoral communities, for example, share grazing lands with wildlife and may require culturally sensitive measures that preserve traditional livelihoods while concurrently reducing disease risks [5, 146]. South American farms bordering protected conservation areas and expanding wild boar populations in Europe present other distinct scenarios, each necessitating tailored interventions that account for ecological, social, and regulatory contexts [147, 160, 161, 172]. Although the United States is currently FMD-free, feral pigs would present a major challenge if the FMDV were introduced [144, 152]. These highly adaptable animals frequently forage in agricultural fields and share water sources with livestock, increasing opportunities for disease transmission. Human-mediated translocations, such as the intentional release of feral pigs for recreational hunting purposes, exacerbate the issue by introducing populations into new areas, often without regulatory oversight [144, 173, 174]. The anthropogenic movement of wild and feral animals disrupts existing management efforts, facilitates the expansion of feral pig ranges, and creates new hotspots for interaction with livestock. The economic burden of feral pig management in the United States is substantial, encompassing damages to agriculture and significant control costs [144, 152, 175].

Addressing these diverse challenges and knowledge gaps will depend on the improved integration of ecological and behavioral data into epidemiological models, as well as the continuous refinement of surveillance and control methods. Future advances may involve harnessing climate models to predict seasonal shifts in wildlife distributions, employing machine-learning approaches to analyze complex host–pathogen data, and incorporating participatory research methods to engage local communities in surveillance and control strategies. Combining advanced diagnostics, ecological management, and community outreach has the potential to improve outbreak forecasting, prioritize targeted interventions, and strike a balance between effective disease control, biodiversity conservation, and the protection of livelihoods [140, 143, 150, 169, 176].

Ultimately, wildlife’s role in FMD epidemiology varies across ecological and geographical contexts, and no single strategy will be universally applicable. However, reducing wildlife’s contribution to the global FMD burden may be possible by refining theoretical frameworks, incorporating emerging technologies, and strengthening local capacity. The insights derived from wildlife research are thus indispensable for developing more resilient, adaptive approaches to control this transboundary pathogen and safeguard animal health, human livelihoods, and ecosystem integrity in ever-changing environmental and socioeconomic landscapes.

## Computational modeling

### Application of computational models

Computational models of disease transmission are increasingly used to estimate the behavior and impacts of FMD. This is true for both endemic and free countries, and the results have provided much information regarding the biological behavior of outbreaks and the options for control. Several national models have been built to estimate impacts and control options to support decision-making and policy in FMD-free countries [177–184].

These models are commonly used to estimate the effect of alternate control strategies and may track both outbreak metrics and economic factors as outputs. A recent model implemented for Denmark estimated the effects of index herd region and production type, detection time, and resource allocation as factors influencing the outcome. However, additional control strategies beyond baseline movement controls, tracing, and depopulation of disease positive premises did not change outcomes [185]. Tsao et al. [186] developed a model for herd-to-herd transmission in cattle only and simulated outbreaks from index herds in all 3049 counties in the contiguous United States. In most scenarios, fewer than ten herds were infected, and demographic variables related to the seed herd size, county incoming shipments, and herd clustering were influential in the final outbreak size. Evaluating a UK farm density-based culling strategy suggested the strategy was more effective than total ring culling when comparing total culled animals, total culled farms, and outbreak duration [187]. Analysis of implementing trading zones in Australia in the face of an FMD outbreak indicated substantial economic benefits [188]. Hafi et al. [188] demonstrated a positive effect of vaccine allocation and additional surveillance resources on outbreak size in Australia, and Sanson et al. [189] demonstrated decreased outbreak size and duration in New Zealand in response to increased numbers of veterinarians available for control efforts. While most modeling for FMD has been focused on between-herd models, some within-herd models of feedlots in the United States have been developed recently to describe disease dynamics and the potential impact of interventions. Beck-Johnson et al. [190] examined alternate parameterizations of the temporal relation between individual animal infectiousness and clinical signs. They found little difference in the total length of time a feedlot was infectious but notable differences in how long it was infectious before detection, highlighting the need for clarity regarding this parameter. Models of feedlots in the United States found within herd duration of infection of 49–82 days [191, 192]. Two models examined the effect of depopulation and or vaccination intervention strategies within an infected feedlot [193, 194], but none of the interventions successfully prevented infection of most cattle pens within the feedlot. The risk of airborne transmission has not been studied extensively; however, a recent review [144] and two papers have evaluated the temporal and geographic risks of transmission [195] and the risk from US feedlots [196].

National models produced for use in FMD-free countries are generally not well-suited for use in endemic countries due to the inability to incorporate multiple circulating FMDV strains, routine prophylactic vaccination, and within-herd population dynamics, as well as fundamental differences in livestock production systems [165]. Models implemented for application in endemic countries generally focus on available funding associated with a particular question. They have, however, provided useful assessments of FMD national or regional behavior and potential control options. Recent individual models applied in endemic countries have assessed the impact of depopulation, ring vaccination, animal movement restrictions, and farm isolation on outbreak control in Thailand [197], cross-border trading quarantine strategies between Thailand and Myanmar [198], mass vaccination programs in India [199], and the impact of human and animal networks on final epidemic size in Cameroon [200, 201]. A spatial kernel model in Vietnam based on previous outbreak data estimated relatively long-distance transmission compared to published estimates [202].

### FMD Risk estimates

Other recent studies have attempted to predict the risk of FMD in endemic countries. Methods used include GIS-based multi-criteria decision analysis in Thailand based on expert-evaluated spatial risks from 2014 to 2015, resulting in moderate agreement with outbreaks in 2016 [203]. Machine learning based on 15 binary predictor variables from a previous case and control herds in an outbreak in Thailand reported accuracy greater than 70% in predicting positive farms, with relatively high specificity (> 80%) but low sensitivity (< 35%) [204]. Machine learning based on low-resolution national data and local serosurvey data in two regions in Myanmar resulted in the incorrect classification of 40% of villages, suggesting that low-resolution national data alone was not sufficient for prediction [205]. Gonzalez Gordon et al. [163] reviewed spatial and spatial–temporal studies to summarize risks associated with endemic outbreaks, identifying animal demographics, transport infrastructure, trade, environment, and socioeconomic factors as important. Serotype O genetic sequence data and phylogenetic techniques applied to field data from Uganda suggest that areas of high cattle density and those areas surrounding livestock markets may serve as FMDV sources, and areas with low rainfall may serve as sinks, resulting in frequent outbreaks [107]. Combining specific geographical data with biological epidemic data may further identify geographical barriers and promoters of disease transmission [167].

Several recent models have evaluated the risk of FMD introduction across borders or within an individual herd. Brusa et al. [206] estimated the risk of FMDV introduction from Argentina, an FMD-free with vaccination nation, to an importing nation through bone-in beef and offal as negligible. McKee et al. [207] estimated the highest risk areas for FMD introduction into the United States through contaminated meat products, and Meyer et al. estimated the risk of FMD introduction to the United States through imports of semen from Israel [208] and released from the United States bull studs under FMD testing protocols [209]. A decision tree risk analysis for Egyptian feedlots identified the physical examination of new introductions and vaccination on arrival as important protective factors [210].

### Parameter impacts

A common problem for all models, and one that becomes increasingly acute as model complexity increases, is the lack of specific and quantitative parameters. Data limitations regarding circulating strains, the evolution of new lineages, strain-specific infectious behavior, livestock demography, livestock-human movement networks, and management system behaviors limit computational models’ reliable application and interpretation [6, 211].

Substantial work has been accomplished in parameter estimates of FMD disease durations, phases, and infection dynamics. However, the available data is limited in strain variability, with serotype O predominantly represented in data [212–215].

Recent studies have described livestock movement networks in free countries [193, 216]; however, substantial uncertainty still surrounds livestock movement networks in countries without mandated collection of movement data, limiting models’ ability to assess network impacts or effective interventions with incomplete or outdated movement data [217].

More robust movement network data in the United States is available for swine and suggests variability in movement patterns across production systems, illustrating the need for comprehensive data [218]. Counties with mandatory livestock movement data collection have a more robust understanding of the movement network to assess impacts. Social network analysis is an effective method to analyze outbreak metrics when data is available. Iriarte et al. [219] used records of livestock movements in Uruguay during 2022 to estimate network impacts on transmission and between farm R-value, showing that livestock markets and highly connected farms were responsible for early, long-distance FMD spread.

Recent work on network analysis has explicitly incorporated the temporal sequence of movements to identify farms with high infection potential [218]. Analysis of agent-based model-generated infection networks supports combinations of node and global level metrics in complex interactions as better predictors of disease risk than individual network metrics [220]. Statistical processes may also generate networks to represent contacts when data is not available. The best method to represent a particular production system is difficult to assess; different methods may impact results [200] and differ from results produced by empirical networks [201, 221]. While indirect contacts may also transmit infection, the authors are unaware of any published network estimates of indirect contacts or resultant infection risk between farms.

Models have also been used to assess the impact of uncertain or variable model parameters on outbreak impact. Gilbertson et al. [222] evaluated the impact of changing assumptions regarding premises transmission behavior within and between herds through livestock shipments. They concluded that modeled transmission behaviors were important but less influential than livestock demographics. Similarly, Kinsley et al. [223] demonstrated that altering assumptions about farm demography and barn/farm structure impacted epidemiological predictions; however, the importance of incorporating these within-farm complexities depended on the outcome of interest (i.e., time to detection, time to peak infection, and long-term persistence on the farm). Meadows et al. [224] evaluated the impact of livestock and farm density on epidemic size, suggesting that livestock density was the more critical factor. Data on farm locations and herd sizes are necessary to correctly represent farm and livestock density. For spatially explicit models, explicit farm locations are required but not always available. In the United States, farm locations are only available at the county level, and individual locations must be simulated for spatially explicit models. The random placement of farms at the county level does not capture local clustering of livestock or farm density, and methods to allow clustering in premises locations are important to improve predictive outcomes [225]. Some models have examined the impact of vaccine effectiveness on outbreak metrics. Han et al. [226] simulated the impact of a mismatched novel FMDV strain introduction to vaccinated swine in South Korea, resulting in an outbreak. The best fit models for the daily clinical data indicated decreased viral shedding but no decrease in clinical signs. Modeling quarantine station risk of FMD release at the Thailand-Myanmar border indicated that vaccine effectiveness is influential [198]. The ability of vaccine strains to produce immunity to the locally circulating strains is important but not explicitly captured in available models.

While computational modeling methods have advanced, the parameterization of available models remains a challenge. Underlying population demographics, geographic locations, and interactions are often unavailable in sufficient detail, yet they are influential in predicting outcomes. Additionally, variability in strain virulence and infectivity are largely unknown, and the relation between individual animal infection and shedding dynamics, within farm transmission and infectivity, and between farm transmission has not been addressed.

## Economics

Economics concerns rational decisions about allocating scarce resources to achieve competing goals. Research is key in providing the evidence and conceptual understanding necessary to inform decisions. Here, recent accomplishments and current research gaps in FMD economics are considered.

FMD is usually listed as the priority livestock disease for countries with developed commercial livestock sectors, where the presence of FMDV would block access to valuable export markets, and regular outbreaks undermine livestock productivity and profitability. FMD is a top-three priority disease for many low and middle-income country (LMIC) governments. However, because of FMD’s generally low mortality in LMIC regions, the disease’s economic impact can be overlooked, with the burden arising from the regular, high-incidence outbreaks of short-term acute illness, but with long-term production loss, particularly in more productive, intensive systems.

Understanding the true economic impact of FMD is particularly important in light of its divergent prioritization across settings. While high-income countries often respond to single incursions with costly eradication efforts, the more chronic and underappreciated burden in endemic LMICs points to the need for models that can quantify both visible and hidden losses across a range of production systems.

Despite notable limitations, economic models offer valuable insights into the magnitude of economic losses incurred during FMD outbreaks. For instance, estimated annual losses in FMD-endemic regions range broadly from $6.5 to $21 billion, primarily reflecting production losses and vaccination costs [141, 227]. Major epidemic outbreaks in historically FMD-free countries illustrate even greater potential impacts; notably, the 2001 outbreak in the United Kingdom resulted in economic losses estimated at approximately $9.2 billion, encompassing direct agricultural costs, control measures, and substantial losses in tourism and related sectors [228]. Outbreaks in Taiwan (1997) and South Korea (2010–2011) resulted in economic damages of roughly $6.6 billion and $2.8 billion, respectively, highlighting the economic vulnerability of export-oriented nations [227]. Control measures alone represent significant expenditures, as demonstrated by the UK’s public sector costs, which approached nearly $4 billion for compensation and operational expenses during the 2001 outbreak [228]. These cases clearly illustrate the significant economic threat posed by FMD and reinforce the importance of developing more accurate economic models to better inform decision-making and resource allocations.

### Economic drivers of control

Before investing in a control strategy, policymakers and livestock keepers need to know the disease burden and consider the benefits of control. They can then compare the return on investing in FMD control with other available investment options and, if funds are available, choose the best investment. For countries with developed livestock systems and appreciable export of livestock commodities, typically middle- or high-income countries with veterinary services capable of controlling FMD, the benefits of eradication and maintenance of free status have historically been taken as a given. A key element is maintaining FMD-free status to access lucrative export markets. This contrasts with most LMICs, where widespread control programs are rarely implemented because of the need for long-term investment to build strong government services that can implement nationwide effective control programs for fast-moving diseases like FMD. This is compounded by the relatively high cost of effective FMD vaccines compared to vaccines for other priority diseases. Furthermore, in extensive livestock systems based on indigenous breeds that are less susceptible to FMD, the direct impact of FMD is lower; however, with a high incidence, the loss can still be considerable.

There are a limited number of studies examining the national impact of FMD, and even fewer that assess the return on potential control policies. However, widespread effective control is beyond the reach of most LMICs, or they have other priorities that would deliver a better and more reliable return on investment. The focus should be on transitioning economies where agricultural intensification is accelerating and public services are increasingly effective, as these are the countries that will benefit the most from FMD control and are able to achieve it.

### Frameworks for assessing FMD impact

Long ago, livestock production and animal health economics seemed like a relatively simple affair, considering the costs of inputs, how disease affects the quantity of outputs, assigning a financial cost to these components, and then comparing different scenarios. However, global needs increasingly go beyond food and finance. The negative impacts of livestock production are increasingly recognized by society, particularly in terms of animal welfare and the effects of livestock production on ecosystems, such as slurry pollution and climate change, with livestock accounting for approximately 9% of anthropogenic Greenhouse Gas (GHG) emissions [229]. Animal health is important for the latter as the intensity of GHG emissions (e.g., units of GHG emissions per unit of output, such as per liter of milk) greatly reduces if animals are more productive. An unproductive cow, for example, still emits GHGs simply by being alive, without gaining weight or producing milk. Improving animal health, along with other production inputs such as nutrition, will increase an animal’s production and production efficiency while reducing GHG emission intensity. Livestock farming also has spillover impacts on the wider environment through contamination, biodiversity reduction, and habitat loss. With more efficient production, the same quantity of food can be produced with a smaller ecological footprint. Another indirect impact of FMD and other diseases is that they result in increased medicine usage and incremental contribution to human and animal antimicrobial resistance.

**Figure 2:**
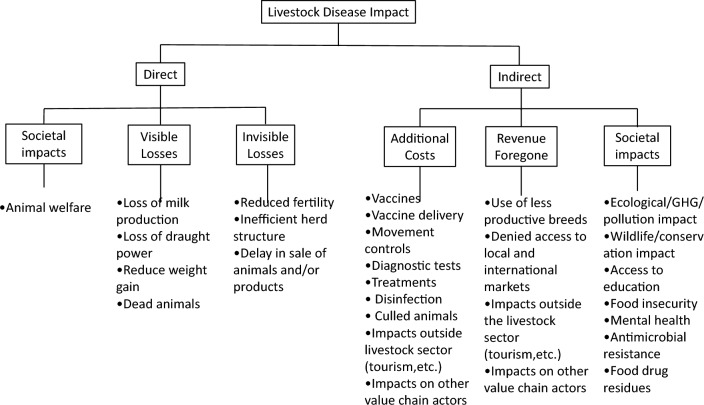
**The impacts of FMD**. Impacts on other value chain actors includes those on butchers, livestock markets, middlemen, providers of inputs etc. Impacts outside the livestock sector include those on industries such as tourism and crop production. GHG: Green House Gas. Adapted from Knight-Jones and Rushton 2013; and Rushton 2009.

These broader impacts are included in the framework below (Figure 2).

### Knowledge of impacts and benefits of control

Studies on FMD impacts, promoted by the Progressive Control Pathway for FMD [230], continue to be published for countries where governments or individual researchers are interested in FMD control [231]. However, although overarching approaches are standardized, each study usually uses its own ad hoc methods within these frameworks. Most available FMD economic studies are not grounded in established practices of animal health economics and are unlikely to provide robust evidence on the use of FMD control resources [232]. As an economic model needs to consider both FMD epidemiology and its economic consequences, there are many opportunities for errors and inappropriate parameter estimates to be incorporated. Often, this is compounded by the lack of good livestock production data. As a result, many FMD economic studies are not robust, and some have significant flaws. An error in a single parameter out of a hundred can propagate through the model, resulting in an estimate that is several multiples of the likely true value.

Unfortunately, it is usually almost impossible for readers to detect these issues as the models are complex and somewhat black-box, and predictions cannot usually be verified. As with all models, one must consider if they fit the purpose and if the estimate is useful. Better standardization of approaches will improve matters.

However, adapting methods to the available data will always require compromises.

Standardized, validated FMD epidemiological models are widely used, particularly to model outbreaks in FMD-free countries [181]. However, an extension of these model platforms to assess economic outcomes is yet to be done. This would be of use for both FMD-free and endemic settings. At the other end of the complexity spectrum, simple cost calculators would also be useful in informing farm-level management decisions about FMD vaccination strategies.

Inspired somewhat by the human Global Burden of Disease work [233], and looking to fill the relative knowledge void for animal health, the Global Burden of Animal Diseases (GBADs) program has developed innovative approaches for estimating the total financial burden of animal diseases by comparing the current situation to a perfect world scenario with no animal disease, with no disease induced reduction in productivity and no premature death or culling [234]. These losses are colossal, accounting for a halving of the value of livestock outputs due to poor health [235]. Although striking, this parallels changes in productivity levels that occurred as Western livestock systems as they intensified over the course of the twentieth century, during which most measures of productivity more than doubled [236]. The burden from all diseases described by the Animal Health Loss Envelope can be attributed to different diseases [237]. This provides an innovative approach; however, there is still a need to collect more accurate routine data so that empirical evidence can inform parameters currently estimated by expert opinion. The economics of control in different settings.

### The economics of control in different settings

The Global FMD Control Strategy aims to reduce the global burden of FMD. As mentioned, the world is highly divided regarding incentives and the capacity to control FMD. Endemic countries are guided in their progress toward FMD control through the PCP-FMD. However, in many endemic countries, whilst the veterinary services may be interested in controlling FMD and often develop the necessary plans and strategies, lacking the necessary resources and capacity, and with complex livestock systems with communal and migratory grazing, many struggle to implement effective FMD control on the ground, even within a subset of the population.

Countries have been supported in doing FMD impact assessments, which are used to justify and advocate for funding for FMD control. For example, EuFMD and FAO have provided training courses in animal health economics, which capacitate countries to do these assessments, with guidelines on economic analysis for FMD control. However, countries must go beyond impact assessments and consider the benefits and costs of a control program. Different control options need to be compared to see which is most promising. If this is done well, untested and ineffective controls, such as those using vaccines with low or unproven quality, are less likely to be performed, further emphasizing the need for quality and cost-effective approaches.

Endemic countries with no or poor FMD control pose a threat to neighboring countries where FMD is controlled. However, economic analyses or control policies rarely capture this externality, where the neighbor experiences some of the benefits of vaccinating. Economic studies are needed to capture this and determine the appropriate level of external funding required to control reservoirs of poor FMD control, thereby reducing the burden on the regional livestock sector. At a local level, the economics of Commodity Based Trade should be better demonstrated to allow sector growth in areas where zonal FMD freedom is difficult because of communal and mobile livestock systems and endemic wildlife.

To inform outbreak strategies in FMD-free countries, the economic rationale for vaccinate-to-live approaches (where freedom is achieved with vaccination, but vaccinated stock are not subsequently culled) must be better understood compared to vaccinate-to-kill approaches, where vaccinated animals are later culled as they pose a greater risk of harboring subclinical infection, which is likely to go undetected.

### Behavioral economics

Behavioral economics combines psychology and economics to understand how and why people behave in certain ways. It is critical for animal disease control, where all value chain actors, particularly the livestock keepers, influence what happens and the choices made regarding livestock disease control. This field is seldom considered in FMD control, even in FMD-free countries with advanced FMD research agendas, maybe because the established strategies predate the field of behavioral economics.

In free countries, livestock keepers are compensated for livestock culled during an eradication campaign. This is critical; if compensation is inadequate, cases are hidden, and they cannot be controlled if they are not detected. Compensation also accounts for a huge proportion of the costs of an eradication campaign, with, for example, over six million animals culled in the UK 2001 FMD outbreak. Despite this, there is little understanding of how compensation can be utilized to maximize the cost-effectiveness of a control campaign. Funding research in this area will be a sound investment for veterinary services, with findings relevant to all diseases. In endemic countries, there is a need to better understand the drivers of livestock keepers’ participation and payment for FMD vaccines and healthcare for their animals in different systems.

Global donors have stated there is a void of information regarding the burden of livestock diseases and the economics of animal disease control. GBADs has looked to address this. National and global estimates are important and indicate priority areas, but decisions about FMD control need to address specific questions and assess specific strategies in specific settings. Assessments should focus on settings where control is feasible and funding is available if control is predicted to be rational. For FMD, this applies to countries with emerging livestock sectors and export potential that want to invest in FMD control but require additional guidance and evidence to direct their control strategy. The assessment of FMD impact and control cost efficiency needs to be based on robust, standardized, and transparent approaches in both the epidemiological and economic aspects to generate useful evidence for the best use of resources in its control.

## Conclusions

Due to its complex epidemiology, diverse host range, and wide-ranging socioeconomic impacts, FMD is a paradigmatic challenge in the management of transboundary animal diseases. This review has explored multiple dimensions of FMD epidemiology, from global patterns and molecular characteristics to pathogenesis, wildlife interactions, computational modeling, and economic factors, revealing interlinked knowledge gaps that limit the effectiveness of current control and mitigation strategies. A comprehensive and detailed breakdown of these gaps is provided in Table 1, which offers a more granular view of the discussed challenges.

**Table 1:** **FMD knowledge gaps**

**Global epidemiological trends**
• Limited understanding of the drivers behind serotype translocations, including the factors influencing the emergence and spread of SAT serotypes into new territories • Limited understanding of the drivers behind intra-serotypic shifts, including the factors influencing the interplay of O topotypes MESA-PanAsia, MESA-Ind2001, and Mya-98 • Limited understanding of how climate change and extreme weather events influence the global and regional distribution of FMD risks, including the spread of serotypes under novel environmental conditions • Insufficient global surveillance networks capable of providing near real-time situational awareness, particularly concerning animal movements, product trade patterns, and molecular epidemiology • Need for improved approaches to estimate the true burden of FMD, reducing reliance on biased “outbreak” data and resource-limited sampling • Lack of effective integration between passive and active surveillance systems, especially in endemic regions, resulting in delayed detection of new strains and hidden transmission pathways • Insufficient analysis of evolving international trade policies and undocumented animal movement dynamics in shaping FMD dissemination pathways • Limited understanding of the factors that led to the presumptive extinction of serotype C and how this might be exploited to control and eradicate other FMDV strains and serotypes
**Pathoepidemiology**
• Incomplete understanding of the influence of subclinical (neoteric) infections on FMDV epidemiology, including the risk posed by the movement of subclinically infected animals. • Incomplete understanding of the contribution of small ruminants to FMDV epidemiology. • Limited knowledge of differences in clinical FMD, viral shedding dynamics, and associated transmission potential of indigenous cattle breeds. • Uncertain role of carrier animals in FMDV transmission and evolution, specifically in relation to heterologous superinfection of persistently infected carriers giving rise to recombinant FMDVs. • Need for improved immunoassays with low cross-serotype reactivity to accurately differentiate vaccinated from infected animals, especially in multi-serotype settings. • Poorly defined relationships between individual animal infection dynamics (e.g., viral shedding profiles) and farm-level or between-farm transmission patterns. • Unclear mechanisms driving the within-host evolution of FMDV during persistent infections, particularly under varying immunological and therapeutic pressures. • Limited research into the impacts of concurrent infections, such as peste des petits ruminants virus, on FMD pathogenesis and host immunity. • Poorly defined thresholds of viral shedding necessary to initiate secondary infections, particularly during preclinical stages when overt signs are absent but transmission risk remains high. • Limited data on how viral load dynamics affect the duration of incubation periods and the progression to clinical or subclinical infections, particularly in diverse host species. • Limited understanding of how host-specific immune responses, including innate and adaptive immunity variations, modulate viral replication, shedding dynamics, and subsequent transmission potential in multispecies contexts. • Poorly defined impact of viral quasispecies diversity within individual hosts on transmission potential, adaptation to new hosts, and persistence in variable ecological and epidemiological conditions.
**Molecular Epidemiology**
• Insufficient genomic data from underrepresented regions and serotypes constrain understanding of global viral diversity • Limited application of integrated genomic-epidemiological analyses to trace outbreak origins, transmission routes, and vaccine effectiveness • Absence of standardized tools for leveraging next-generation sequencing (NGS) sub-consensus data to improve viral lineage tracing • Lack of hybrid frameworks integrating phylogenetic and epidemiological data to trace the dynamics of cross-species viral transmission and emergence • Poor understanding of the evolutionary pressures shaping serotype shifts within ecological and epidemiological constraints • Need for more cost-effective and widely accessible methods to collect, process, and analyze viral sequences from diverse FMD contexts
*Wildlife interactions*
• Poor understanding of the roles played by lesser-studied wildlife species in maintaining and transmitting FMD • Limited incorporation of wildlife-specific behaviors, ecology, and seasonal resource variability into epidemiological and modeling frameworks • Poor understanding of the roles played by marginal species or wildlife species considered secondary reservoirs but may intermittently contribute to disease dynamics in specific ecological contexts • Limited exploration of how habitat fragmentation, resource competition, and human encroachment modify wildlife-livestock interfaces, particularly in endemic regions • Underdeveloped surveillance systems for wildlife in FMD-free regions, hindering early detection of potential incursions via migratory species or bridge hosts • Insufficient knowledge of the risks posed by migratory species in acting as mechanical carriers or vectors for FMD introduction across borders, especially in areas with limited veterinary infrastructure • Lack of predictive modeling to assess how shifts in wildlife population densities or migratory patterns influence FMD risk under changing climatic or land-use conditions • Underdeveloped wildlife surveillance systems, including inadequate use of non-invasive methods such as environmental DNA (eDNA) sampling
**Computational modeling**
• Significant parameterization challenges due to limited data on livestock demographics, movement networks, management practices, and strain-specific viral characteristics • Inability of current models to evaluate real-time cost-effectiveness of control, surveillance, and sampling strategies during outbreaks • Underutilization of advanced, integrated models incorporating social, economic, and ecological parameters to predict disease dynamics and intervention outcomes
**Economics**
• Absence of standardized frameworks to integrate epidemiological data with economic analyses, hindering the evaluation of control measure cost-effectiveness • Limited consideration of behavioral economic factors, such as livestock keeper decision-making and the impact of compensation schemes on reporting and compliance
**Diagnostics and surveillance**
• Lack of validated, field-ready diagnostic tools (e.g., rapid, pen-side assays) for timely FMD detection and outbreak response • Challenges differentiating vaccinated from infected animals using current serological methods complicate surveillance and movement controls
**Cross-cutting gaps**
• Need for robust epidemiological models incorporating economic outcomes applicable to endemic and FMD-free settings • Need for data-driven shedding and transmission parameters spanning distinct species, breeds, and viral genotypes to improve downstream simulation modeling • Limited application of advanced analytical tools, such as genome-wide association studies and machine learning, to identify key viral maintenance and spread drivers

Analysis of global epidemiological trends highlights dynamic shifts in serotype prevalence, notably the expansion of serotype O Ind2001 viruses and the emergence of SAT serotypes in new territories. These changes indicate a knowledge gap in understanding how intrinsic viral properties interact with the host and environment to determine the ultimate fitness of a virus and its ability to endure when introduced to new regions. Further, there are gaps in adaptable surveillance systems, with a need for improved early detection in FMD-free regions and deeper insights into disease maintenance mechanisms within endemic settings. The gaps table (see “Global Epidemiological Trends” in Table 1) identifies specific deficits, such as limited understanding of serotype geographic shifts and insufficient integration of passive and active surveillance, offering concrete targets for future research.

The complexity of FMD pathogenesis, including neoteric subclinical infections, persistently infected carriers, and species-specific susceptibilities, adds another layer of difficulty in elucidating FMDV epidemiology mechanisms, ultimately compromising detection and control. Similarly, advances in molecular epidemiology underscore the importance of robust genomic tools for tracing transboundary movements and informing vaccine strain selection. Yet, a significant underrepresentation of specific regions and serotypes persists. The gaps related to “Pathogenesis” and “Molecular Epidemiology” (Table 1) illustrate these issues in detail, highlighting the need for improved immunoassays, more comprehensive genomic data collection, and standardized methods for analyzing NGS-derived information. Improved tools are needed to analyze ever-increasing pools of FMDV “big data” genomics, including full-length sequence analysis and the relevance of deep sequence quasispecies variation in population-level viral fitness.

Computational modeling has emerged as a powerful yet underutilized tool, hindered by challenges in parameterization, limited data availability, and difficulties in integrating wildlife and ecological factors. Several identified modeling gaps, such as insufficient livestock demographic data and the limited application of advanced, integrated models, are listed in the “Computational Modeling” section of (Table 1). These gaps suggest that real-time decision-support tools capable of evaluating epidemiological and economic outcomes could bridge the divide between theoretical modeling and on-the-ground disease management.

The role of wildlife in maintaining and spreading FMD remains insufficiently understood, and more robust wildlife surveillance systems, ecological data integration, and non-invasive diagnostic approaches are needed. Gaps highlighted under “Wildlife Interactions” detail the lack of tailored models for wildlife-specific behaviors, limited understanding of lesser-studied species, and inadequate methods to leverage emerging techniques such as environmental DNA sampling. Addressing these wildlife-related gaps can support strategies that balance disease control with biodiversity conservation.

The economic aspects of FMD control underscore persistent inequities in resource allocation and highlight the complexity of translating epidemiological insights into cost-effective interventions. Limited evidence exists on the cost-effectiveness of different strategies, the influence of behavioral economics, and the integration of socioeconomic data. The “Economic Impacts” and “Cross-Cutting Gaps” entries in Table 1 highlight the importance of standardized frameworks that connect epidemiological data with economic analyses, guiding rational decision-making for diverse stakeholders and production systems.

Among the identified research gaps, three stand out as particularly critical due to their cross-cutting relevance and direct implications for surveillance, modeling, and control strategies. First, the role of subclinical, particularly neoteric, infections remains poorly understood yet central to FMDV epidemiology. These often undetected infections can silently drive transmission across herds, populations, and regions, especially in endemic settings where passive surveillance of clinical cases may miss early signs or mild cases. Addressing this gap will require a combination of field-based studies and controlled animal experiments designed to characterize the duration, shedding dynamics, and transmission potential of neoteric infections.

Experimental studies across species, viral strains, and exposure routes would enable a more precise delineation of early infection stages and improve detection tools for preclinical or subclinical carriers.

Second, the lack of genomic data from underrepresented regions and serotypes hinders the understanding of FMDV evolution and global spread. This gap limits the resolution of phylogeographic analyses, impedes real-time outbreak tracing, and restricts vaccine matching efforts in highly diverse or antigenically shifting contexts. Expanding sequencing capacity would not only improve outbreak response but also deepen the understanding of FMDV biogeography as it relates to international linkages between disease reservoirs, livestock movement networks, and regional viral persistence. Comprehensive genome collection and data integration are crucial for mapping transmission corridors, identifying priority areas for surveillance, and assessing the risk of introduction into FMD-free regions.

Third, parameterization challenges continue to constrain the realism and utility of epidemiological models, particularly due to limited data on livestock demographics, transportation patterns, production systems, and strain-specific viral characteristics. In addition to controlled animal experiments that can provide empirically verified transmission parameters, this priority also calls for more direct engagement with farmers, producers, and veterinary networks. Surveys, interviews, and participatory workshops can help researchers understand real-world behaviors, decision-making processes, and existing biosecurity infrastructure. This type of stakeholder-informed data is critical for building realistic models that incorporate plausible intervention scenarios, behavioral responses, and even game-theoretic considerations. Improved communication between scientists and stakeholders leads to more credible models and more effective, locally relevant disease control strategies.

Addressing the knowledge gaps identified herein will require sustained, equitable, and inclusive efforts that reflect the differing needs and realities of endemic and FMD-free regions. Cross-referencing the tabulated gaps with the high-level priorities can support interdisciplinary collaboration and ensure that research investments are both strategic and context-specific. Coordinated approaches that integrate advanced diagnostics, ecological and molecular surveillance, behavioral economics, and participatory engagement with affected communities are essential for mitigating transmission risks and enhancing global FMD preparedness. By aligning broad strategic priorities with specific research needs, which range from experimental studies and genomic mapping to stakeholder-informed modeling, policymakers and scientists can more effectively prioritize efforts, allocate resources, and implement policies that support long-term, sustainable FMD control and, ultimately, eradication. 

## References

[1] Grubman MJ, Baxt B (2004) Foot-and-mouth disease. Clin Microbiol Rev 17:465–493 15084510 10.1128/CMR.17.2.465-493.2004 PMC387408

[2] Weaver GV, Domenech J, Thiermann AR, Karesh WB (2013) Foot-and-mouth disease: a look from the wild side. J Wildl Dis 49:759–785 24502706 10.7589/2012-11-276

[3] Paton DJ, Sumption KJ, Charleston B (2009) Options for control of foot-and-mouth disease: knowledge, capability and policy. Philos Trans R Soc Lond B Biol Sci 364:2657–2667 19687036 10.1098/rstb.2009.0100 PMC2865093

[4] Paton DJ, Di Nardo A, Knowles NJ, Wadsworth J, Pituco EM, Cosivi O, Rivera AM, Kassimi LB, Brocchi E, De Clercq K, Carrillo C, Maree FF, Singh RK, Vosloo W, Park MK, Sumption KJ, Ludi AB, King DP (2021) The history of foot-and-mouth disease virus serotype C: the first known extinct serotype? Virus Evol 7:veab009 35186323 10.1093/ve/veab009 PMC8102019

[5] Casey-Bryars M, Reeve R, Bastola U, Knowles NJ, Auty H, Bachanek-Bankowska K, Fowler VL, Fyumagwa R, Kazwala R, Kibona T, King A, King DP, Lankester F, Ludi AB, Lugelo A, Maree FF, Mshanga D, Ndhlovu G, Parekh K, Paton DJ, Perry B, Wadsworth J, Parida S, Haydon DT, Marsh TL, Cleaveland S, Lembo T (2018) Waves of endemic foot-and-mouth disease in eastern Africa suggest feasibility of proactive vaccination approaches. Nat Ecol Evo 2:1449–1457 10.1038/s41559-018-0636-x 30082738

[6] Di Nardo A, Ferretti L, Wadsworth J, Mioulet V, Gelman B, Karniely S, Scherbakov A, Ziay G, Özyörük F, Parlak U (2021) Evolutionary and ecological drivers shape the emergence and extinction of foot-and-mouth disease virus lineages. Mol Biol Evol 38:4346–4361 34115138 10.1093/molbev/msab172 PMC8476141

[7] Bachanek-Bankowska K, Di Nardo A, Wadsworth J, Mioulet V, Pezzoni G, Grazioli S, Brocchi E, Kafle SC, Hettiarachchi R, Kumarawadu PL, Eldaghayes IM, Dayhum AS, Meenowa D, Sghaier S, Madani H, Abouchoaib N, Hoang BH, Vu PP, Dukpa K, Gurung RB, Tenzin S, Wernery U, Panthumart A, Seeyo KB, Linchongsubongkoch W, Relmy A, Bakkali-Kassimi L, Scherbakov A, King DP, Knowles NJ (2018) Reconstructing the evolutionary history of pandemic foot- and-mouth disease viruses: the impact of recombination within the emerging O/ME-SA/Ind-2001 lineage. Sci Rep 8:14693 30279570 10.1038/s41598-018-32693-8 PMC6168464

[8] Jamal SM, Khan S, Knowles NJ, Wadsworth J, Hicks HM, Mioulet V, Bin-Tarif A, Ludi AB, Shah SAA, Abubakar M, Manzoor S, Afzal M, Eschbaumer M, King DP, Belsham GJ (2021) Foot-and-mouth disease viruses of the O/ME-SA/Ind- 2001e sublineage in Pakistan. Transbound Emerg Dis 68:3126–3135 33915027 10.1111/tbed.14134

[9] Palinski RM, Bertram MR, Vu LT, Pauszek SJ, Hartwig EJ, Smoliga GR, Stenfeldt C, Fish IH, Hoang BH, Phuong NT, Hung VV, Vu PP, Dung NK, Dong PV, Tien NN, Tho ND, Dung DH, Arzt J (2019) First genome sequence of foot-and-mouth disease virus serotype O sublineage Ind 2001e from Southern Vietnam. Microbiol Resour Announc 8:e01424-e1518 30863819 10.1128/MRA.01424-18 PMC6406109

[10] Ryoo S, Kang H, Lim DR, Kim JM, Won Y, Kim JY, King DP, Di Nardo A, Cha SH (2024) Re-emergence of foot-and-mouth disease in the Republic of Korea caused by the O/ME-SA/Ind-2001e lineage. Front Vet Sci 11:1378769 38689851 10.3389/fvets.2024.1378769 PMC11060149

[11] Nikiforov V, Shcherbakov A, Chvala I, Kremenchugskaya S, Korennoy F, Mayorova T, Timina A, Tyulegenov S, Abdrakhmanov S, Berdikulov M, Sainnokhoi T, Gombo-Ochir D, Tserenchimed T, Prokhvatilova L, Sprygin A (2023) Insights into the molecular epidemiology of foot-and-mouth disease virus in Russia, Kazakhstan, and Mongolia in terms of O/ME-SA/Ind-2001e sublineage expansion. Viruses 15:598 36992307 10.3390/v15030598 PMC10056362

[12] Zainuddin N, Susila EB, Wibawa H, Daulay RSD, Wijayanti PE, Fitriani D, Hidayati DN, Idris S, Wadsworth J, Polo N, Hicks HM, Mioulet V, Knowles NJ, King DP (2023) Genome sequence of a foot-and-mouth disease virus detected in Indonesia in 2022. Microbiol Resour Announc 12:e0108122 36622181 10.1128/mra.01081-22 PMC9933659

[13] Tyulegenov S, Zhakupbayev A, Berdikulov M, Karibayev T, Yessembekova G, Sultanov A, Perez A, Abdrakhmanov S (2022) Foot-and-mouth disease in Kazakhstan. Transbound Emerg Dis 69:1712–1714 35678092 10.1111/tbed.14607

[14] Nardo AD, Shaw AE, Gondard M, Wadsworth J, Girault G, Parekh K, Ludi A, Mioulet V, Bernelin-Cottet C, Hicks HM, Polo N, Bulut A, Parlak U, Gizaw D, Ababneh M, Ameer MA, Abdulrasool LMS, Saloom FSA, Al-Rawahi WA, Knowles NJ, Bakkali-Kassimi L, King DP (2025) Eastern Africa origin of SAT2 topotype xiv foot-and-mouth disease virus outbreaks, western Asia, 2023. Emerg Infect Dis 31:368–372 39983696 10.3201/eid3102.240395 PMC11845145

[15] Ularamu HG, Ibu JO, Wood BA, Abenga JN, Lazarus DD, Wungak YS, Knowles NJ, Wadsworth J, Mioulet V, King DP, Shamaki D, Adah MI (2017) Characterization of foot-and-mouth disease viruses collected in Nigeria between 2007 and 2014: evidence for epidemiological links between West and East Africa. Transbound Emerg Dis 64:1867–1876 27718336 10.1111/tbed.12584

[16] Canini L, Blaise-Boisseau S, Nardo AD, Shaw AE, Romey A, Relmy A, Bernelin- Cottet C, Salomez A, Haegeman A, Ularamu H, Madani H, Ouoba BL, Zerbo HL, Souare ML, Boke CY, Eldaghayes I, Dayhum A, Ebou MH, Abouchoaib N, Sghaier S, Lefebvre D, DeClercq K, Milouet V, Brocchi E, Pezzoni G, Nfon C, King D, Durand B, Knowles N, Kassimi LB, Benfrid S (2022) Identification of diffusion routes of O/EA-3 topotype of foot-and-mouth disease virus in Africa and Western Asia between 1974 and 2019 – a phylogeographic analysis. Transbound Emerg Dis 69:e2230–e2239 35435315 10.1111/tbed.14562 PMC9795992

[17] Pezzoni G, Bregoli A, Grazioli S, Barbieri I, Madani H, Omani A, Sadaoui H, Bouayed N, Wadsworth J, Bachanek-Bankowska K, Knowles NJ, King DP, Brocchi E (2019) Foot-and-mouth disease outbreaks due to an exotic virus serotype A lineage (A/AFRICA/G-IV) in Algeria in 2017. Transbound Emerg Dis 66:7–13 30222914 10.1111/tbed.13017

[18] Soltan MA, Mahmoud MM, Hegazy Y, Abd-Eldiam MM (2022) Emergence of foot-and-mouth disease virus, serotype O, Europe-South America topotype in Egypt, 2022. Transbound Emerg Dis 69:2409–2411 35679058 10.1111/tbed.14612

[19] Hagag NM, Hassan AM, Zaher MR, Elnomrosy SM, Shemies OA, Hussein HA, Ahmed ES, Ali MH, Ateay M, Abdel-Hakim MA, Habashi AR, Eid S, El Zowalaty ME, Shahein MA (2023) Molecular detection and phylogenetic analysis of newly emerging foot-and-mouth disease virus type A, Lineage EURO-SA in Egypt in 2022. Virus Res 323:198960 36209919 10.1016/j.virusres.2022.198960 PMC10194312

[20] Banda F, Shilongo A, Hikufe EH, Khaiseb S, Kabajani J, Shikongo B, Set P, Kapapero JK, Shoombe KK, Zaire G, Kabilika S, Quan M, Fana EM, Mokopasetso M, Hyera JMK, Wadsworth J, Knowles NJ, Nardo AD, King DP (2022) The first detection of a serotype O foot-and-mouth disease virus in Namibia. Transbound Emerg Dis 69:e3261–e3267 35416412 10.1111/tbed.14561 PMC9790293

[21] Jones R, King DP, Busin V (2025) Retrospective analysis of submissions to the world reference laboratory for foot-and-mouth disease: what can these data tell us about the role of small ruminants in disease epidemiology? Prev Vet Med 239:106526 40174344 10.1016/j.prevetmed.2025.106526

[22] Stenfeldt C, Arzt J (2020) The carrier conundrum: a review of recent advances and persistent gaps regarding the carrier state of foot-and-mouth disease virus. Pathogens 9:167 32121072 10.3390/pathogens9030167 PMC7157498

[23] Kabir A, Kamboh AA, Abubakar M, Baloch H, Nizamani ZA (2024) Foot-and-mouth disease virus dynamics in border areas of Pakistan with Afghanistan. Mol Biol Rep 51:370 38411732 10.1007/s11033-024-09262-6

[24] Ludi AB, Morris A, Gubbins S, Asfor A, Afzal M, Browning CF, Grazioli S, Foglia EA, Wilsden G, Burman A, Brocchi E, Paton DJ, King DP (2022) Cross-serotype reactivity of ELISAs used to detect antibodies to the structural proteins of foot-and-mouth disease virus. Viruses 14:1495 35891476 10.3390/v14071495 PMC9316314

[25] Stenfeldt C, Diaz-San Segundo F, De Los ST, Rodriguez LL, Arzt J (2016) The pathogenesis of foot-and-mouth disease in pigs. Front Vet Sci 3:41 27243028 10.3389/fvets.2016.00041 PMC4876306

[26] Arzt J, Juleff N, Zhang Z, Rodriguez LL (2011) The pathogenesis of foot-and-mouth disease I: Viral pathways in cattle. Transbound Emerg Dis 58:291–304 21366894 10.1111/j.1865-1682.2011.01204.x

[27] Hedger R (1972) Foot-and-mouth disease and the African buffalo (S*yncerus caffer)*. J Comp Pathol 82:19–28 4336115 10.1016/0021-9975(72)90022-9

[28] Perez-Martin E, Beechler B, Zhang F, Scott K, de Klerk-Lorist LM, Limon G, Dugovich B, Gubbins S, Botha A, Hetem R, van Schalkwyk L, Juleff N, Maree FF, Jolles A, Charleston B (2022) Viral dynamics and immune responses to foot-and-mouth disease virus in African buffalo (S*yncerus caffer)*. Vet Res 53:63 35927724 10.1186/s13567-022-01076-3 PMC9351118

[29] Huang CC, Lin YL, Huang TS, Tu WJ, Lee SH, Jong MH, Lin SY (2001) Molecular characterization of foot-and-mouth disease virus isolated from ruminants in Taiwan in 1999–2000. Vet Microbiol 81:193–205 11390103 10.1016/s0378-1135(01)00308-x

[30] Kitching R, Hughes G (2002) Clinical variation in foot-and-mouth disease: sheep and goats. Rev Sci Tech 21:505–512 12523691 10.20506/rst.21.3.1342

[31] Geering WA (1967) Foot-and-mouth disease in sheep. Aust Vet J 43:485–489

[32] Stenfeldt C, Pacheco JM, Singanallur NB, Vosloo W, Rodriguez LL, Arzt J (2019) Virulence beneath the fleece; a tale of foot-and-mouth disease virus pathogenesis in sheep. PLoS One 14:e0227061 31891626 10.1371/journal.pone.0227061 PMC6938329

[33] Ryan E, Horsington J, Durand S, Brooks H, Alexandersen S, Brownlie J, Zhang Z (2008) Foot-and-mouth disease virus infection in young lambs: pathogenesis and tissue tropism. Vet Microbiol 127:258–274 17942248 10.1016/j.vetmic.2007.08.029

[34] Ryan E, Zhang Z, Brooks H, Horsington J, Brownlie J (2007) Foot-and-mouth disease virus crosses the placenta and causes death in fetal lambs. J Comp Pathol 136:256–265 17459409 10.1016/j.jcpa.2007.03.001

[35] Wolf TE, Lazarus DD, Opperman P, Heath L, Ganswindt A, Fosgate GT (2020) Impact of foot-and-mouth disease on goat behaviour after experimental infection with serotype SAT1 virus. Prev Vet Med 176:104912 32066026 10.1016/j.prevetmed.2020.104912

[36] Mansley LM, Dunlop PJ, Whiteside SM, Smith RGH (2003) Early dissemination of foot-and-mouth disease virus through sheep marketing in February 2001. Vet Rec 153:43–50 12885212 10.1136/vr.153.2.43

[37] Farooq U, Ahmed Z, Naeem K, Bertram M, Brito B, Stenfeldt C, Pauszek SJ, LaRocco M, Rodriguez L, Arzt J (2018) Characterization of naturally occurring, new and persistent subclinical foot-and-mouth disease virus infection in vaccinated Asian buffalo in Islamabad Capital Territory, Pakistan. Transbound Emerg Dis 65:1836–1850 30035376 10.1111/tbed.12963

[38] Stenfeldt C, Eschbaumer M, Rekant SI, Pacheco JM, Smoliga GR, Hartwig EJ, Rodriguez LL, Arzt J (2016) The foot-and-mouth disease carrier state divergence in cattle. J Virol 90:6344–6364 27147736 10.1128/JVI.00388-16 PMC4936139

[39] Stenfeldt C, Pacheco JM, Smoliga GR, Bishop E, Pauszek SJ, Hartwig EJ, Rodriguez LL, Arzt J (2016) Detection of foot-and-mouth disease virus RNA and capsid protein in lymphoid tissues of convalescent pigs does not indicate existence of a carrier state. Transbound Emerg Dis 63:152–164 24943477 10.1111/tbed.12235

[40] Sutmoller P, Gaggero A (1965) Foot-and-mouth disease carriers. Vet Rec 77:968–969 5890082 10.1136/vr.77.33.968

[41] Sutmoller P, McVicar J, Cottral G (1968) The epizootiological importance of foot-and-mouth disease carriers. I. Experimentally produced foot-and-mouth disease carriers in susceptible and immune cattle. Arch Gesamte Virusforsch 23:227–235 5680590 10.1007/BF01241895

[42] Stenfeldt C, Hartwig EJ, Smoliga GR, Palinski R, Silva EB, Bertram MR, Fish IH, Pauszek SJ, Arzt J (2018) Contact challenge of cattle with foot-and-mouth disease virus validates the role of the nasopharyngeal epithelium as the site of primary and persistent infection. mSphere 3:e00493-18 30541776 10.1128/mSphere.00493-18 PMC6291620

[43] Van Bekkum J, Frenkel H, Frederiks H, Frenkel S (1959) Observations on the carrier state of cattle exposed to foot-and-mouth disease virus. Tijdschr Diergeneeskd 84:1159–1164

[44] Aggarwal N, Zhang Z, Cox S, Statham R, Alexandersen S, Kitching R, Barnett P (2002) Experimental studies with foot-and-mouth disease virus, strain O, responsible for the 2001 epidemic in the United Kingdom. Vaccine 20:2508–2515 12057606 10.1016/s0264-410x(02)00178-0

[45] Burrows R (1968) The persistence of foot-and-mouth disease virus in sheep. Epidemiol Infect 66:633–640 10.1017/s0022172400028369 PMC2130666 4303955

[46] Stenfeldt C, Lohse L, Belsham GJ (2013) The comparative utility of oral swabs and probang samples for detection of foot-and-mouth disease virus infection in cattle and pigs. Vet Microbiol 162:330–337 23022683 10.1016/j.vetmic.2012.09.008

[47] Tenzin DA, Vernooij H, Bouma A, Stegeman A (2008) Rate of foot-and- mouth disease virus transmission by carriers quantified from experimental data. Risk Anal 28:303–309 18419650 10.1111/j.1539-6924.2008.01020.x

[48] Bertram MR, Vu LT, Pauszek SJ, Brito BP, Hartwig EJ, Smoliga GR, Hoang BH, Phuong NT, Stenfeldt C, Fish IH, Hung VV, Delgado A, VanderWaal K, Rodriguez LL, Long NT, Dung DH, Arzt J (2018) Lack of transmission of foot-and-mouth disease virus from persistently infected cattle to naive cattle under field conditions in Vietnam. Front Vet Sci 5:174 30101147 10.3389/fvets.2018.00174 PMC6072850

[49] Parthiban ABR, Mahapatra M, Gubbins S, Parida S (2015) Virus excretion from foot-and-mouth disease virus carrier cattle and their potential role in causing new outbreaks. PLoS One 10:e0128815 26110772 10.1371/journal.pone.0128815 PMC4482020

[50] Ilott MC, Salt JS, Gaskell RM, Kitching RP (1997) Dexamethasone inhibits virus production and the secretory IgA response in oesophageal–pharyngeal fluid in cattle persistently infected with foot-and-mouth disease virus. Epidemiol Infect 118:181–187 9129595 10.1017/s0950268896007376 PMC2808777

[51] Arzt J, Fish IH, Bertram MR, Smoliga GR, Hartwig EJ, Pauszek SJ, Holinka- Patterson L, Diaz-San Segundo FC, Sitt T, Rieder E, Stenfeldt C (2021) Simultaneous and staggered foot-and-mouth disease virus coinfection of cattle. J Virol 95:e01650-21 34586864 10.1128/JVI.01650-21 PMC8610595

[52] Gibson C, Donaldson A (1986) Exposure of sheep to natural aerosols of foot-and-mouth disease virus. Res Vet Sci 41:45–49 3020658

[53] Donaldson A, Gibson C, Oliver R, Hamblin C, Kitching R (1987) Infection of cattle by airborne foot-and-mouth disease virus: minimal doses with o1 and sat 2 strains. Res Vet Sci 43:339–346 2832913

[54] Arzt J, Pacheco JM, Rodriguez LL (2010) The early pathogenesis of foot- and-mouth disease in cattle after aerosol inoculation: identification of the nasopharynx as the primary site of infection. Vet Pathol 47:1048–1063 20587691 10.1177/0300985810372509

[55] Stenfeldt C, Pacheco JM, Singanallur NB, Ferreira HCDC, Vosloo W, Rodriguez LL, Arzt J (2015) Clinical and virological dynamics of a serotype O 2010 South East Asia lineage foot-and-mouth disease virus in sheep using natural and simulated natural inoculation and exposure systems. Vet Microbiol 178:50–60 25937316 10.1016/j.vetmic.2015.04.004

[56] Donaldson A, Alexandersen S (2001) Relative resistance of pigs to infection by natural aerosols of FMD virus. Vet Rec 148:600 11386447 10.1136/vr.148.19.600

[57] Stenfeldt C, Pacheco J, Rodriguez L, Arzt J (2014) Infection dynamics of foot- and-mouth disease virus in pigs using two novel simulated-natural inoculation methods. Res Vet Sci 96:396–405 24548596 10.1016/j.rvsc.2014.01.009

[58] Stenfeldt C, Pacheco JM, Rodriguez LL, Arzt J (2014) Early events in the pathogenesis of foot-and-mouth disease in pigs; identification of oropharyngeal tonsils as sites of primary and sustained viral replication. PLoS One 9:e106859 25184288 10.1371/journal.pone.0106859 PMC4153717

[59] Stenfeldt C, Bertram MR, Meek HC, Hartwig EJ, Smoliga GR, Niederwerder MC, Diel DG, Dee SA, Arzt J (2022) The risk and mitigation of foot-and- mouth disease virus infection of pigs through consumption of contaminated feed. Transbound Emerg Dis 69:72–87 34237198 10.1111/tbed.14230

[60] Stenfeldt C, Pacheco JM, Brito BP, Moreno-Torres KI, Branan MA, Delgado AH, Rodriguez LL, Arzt J (2016) Transmission of foot-and-mouth disease virus during the incubation period in pigs. Front Vet Sci 3:105 27917386 10.3389/fvets.2016.00105 PMC5116750

[61] Orsel K, Bouma A, Dekker A, Stegeman JA, de Jong MCM (2009) Foot-and-mouth disease virus transmission during the incubation period of the disease in piglets, lambs, calves, and dairy cows. Prev Vet Med 88:158–163 18929417 10.1016/j.prevetmed.2008.09.001

[62] Orsel K, De Jong M, Bouma A, Stegeman J, Dekker A (2007) Foot-and-mouth disease virus transmission among vaccinated pigs after exposure to virus shedding pigs. Vaccine 25:6381–6391 17658199 10.1016/j.vaccine.2007.06.010

[63] Pacheco JM, Tucker M, Hartwig E, Bishop E, Arzt J, Rodriguez LL (2012) Direct contact transmission of three different foot-and-mouth disease virus strains in swine demonstrates important strain-specific differences. Vet J 193:456–463 22342891 10.1016/j.tvjl.2012.01.012

[64] Van Roermund H, Eble P, De Jong M, Dekker A (2010) No between-pen transmission of foot-and-mouth disease virus in vaccinated pigs. Vaccine 28:4452–4461 20416264 10.1016/j.vaccine.2010.04.019

[65] Pacheco JM, Mason PW (2010) Evaluation of infectivity and transmission of different Asian foot-and-mouth disease viruses in swine. J Vet Sci 11:133–142 20458154 10.4142/jvs.2010.11.2.133 PMC2873813

[66] Donaldson A, Alexandersen S, Sørensen J, Mikkelsen T (2001) Relative risks of the uncontrollable (airborne) spread of FMD by different species. Vet Rec 148:602–604 11386448 10.1136/vr.148.19.602

[67] Donaldson AI, Herniman KAJ, Parker J, Sellers RF (1970) Further investigations on the airborne excretion of foot-and-mouth disease virus. Epidemiol Infect 68:557–564 10.1017/s0022172400042480 PMC2130857 4321594

[68] Gloster J (1981) Forecasting the airborne spread of foot-and-mouth disease. Vet Rec 17:370–374 10.1136/vr.108.17.370 7292902

[69] Klein J, Hussain M, Ahmad M, Afzal M, Alexandersen S (2008) Epidemiology of foot-and-mouth disease in Landhi dairy colony, Pakistan, the world’s largest buffalo colony. J Virol 5:53 10.1186/1743-422X-5-53 PMC2386124 18445264

[70] Jamal SM, Ferrari G, Hussain M, Nawroz AH, Aslami AA, Khan E, Murvatulloev S, Ahmed S, Belsham GJ (2012) Detection and genetic characterization of foot-and-mouth disease viruses in samples from clinically healthy animals in endemic settings. Transbound Emerg Dis 59:429–440 22212855 10.1111/j.1865-1682.2011.01295.x

[71] de Carvalho Ferreira HC, Pauszek SJ, Ludi A, Huston CL, Pacheco JM, Le VT, Nguyen PT, Bui HH, Nguyen TD, Nguyen T, Nguyen TT, Ngo LT, Do DH, Rodriguez L, Arzt J (2017) An integrative analysis of foot-and-mouth disease virus carriers in Vietnam achieved through targeted surveillance and molecular epidemiology. Transbound Emerg Dis 64:547–563 26301461 10.1111/tbed.12403

[72] Omondi GP, Alkhamis MA, Obanda V, Gakuya F, Sangula A, Pauszek S, Perez A, Ngulu S, van Aardt R, Arzt J, VanderWaal K (2019) Phylogeographical and cross-species transmission dynamics of SAT1 and SAT2 foot-and-mouth disease virus in Eastern Africa. Mol Ecol 28:2903–2916 31074125 10.1111/mec.15125

[73] Orsel K, Dekker A, Bouma A, Stegeman J, De Jong M (2007) Quantification of foot-and-mouth disease virus excretion and transmission within groups of lambs with and without vaccination. Vaccine 25:2673–2679 17254674 10.1016/j.vaccine.2006.11.048

[74] Bravo De Rueda C, Dekker A, Eblé PL, De Jong MCM (2015) Vaccination of cattle only is sufficient to stop FMDV transmission in mixed populations of sheep and cattle. Epidemiol Infect 143:2279–2286 25464822 10.1017/S0950268814003033 PMC9150967

[75] Ehizibolo DO, Haegeman A, De Vleeschauwer AR, Umoh JU, Kazeem HM, Okolocha EC, Van Borm S, De Clercq K (2017) Foot-and-mouth disease virus serotype SAT1 in cattle, Nigeria. Transbound Emerg Dis 64:683–690 28224715 10.1111/tbed.12629

[76] Blignaut B, Heerden J, Reininghaus B, Fosgate GT, Heath L (2020) Characterization of SAT2 foot-and-mouth disease 2013/2014 outbreak viruses at the wildlife–livestock interface in South Africa. Transbound Emerg Dis 67:1595–1606 31984622 10.1111/tbed.13493

[77] Ranaweera LT, Wijesundara UK, Jayarathne HSM, Knowles N, Wadsworth J, Mioulet V, Adikari J, Weebadde C, Sooriyapathirana SS (2019) Characterization of the FMDv-serotype-o isolates collected during 1962 and 1997 discloses new topotypes, CEY-1 and WCSA-1, and six new lineages. Sci Rep 9:14526 31601911 10.1038/s41598-019-51120-0 PMC6787213

[78] Dhikusooka MT, Ayebazibwe C, Namatovu A, Belsham GJ, Siegismund HR, Wekesa SN, Balinda SN, Muwanika VB, Tjørnehøj K (2016) Unrecognized circulation of SAT 1 foot-and-mouth disease virus in cattle herds around Queen Elizabeth National Park in Uganda. BMC Vet Res 12:5 26739166 10.1186/s12917-015-0616-1 PMC4704403

[79] Hemida MG, Rizk W, EL-Ghareeb, F Al-Hizab, A Ibrahim, (2018) Foot-and-mouth disease virus O/ME-SA/IND 2001 lineage outbreak in vaccinated Holstein Friesian cattle in Saudi Arabia in 2016. Vet Q 38:88–98 30706772 10.1080/01652176.2018.1539568 PMC6831000

[80] Kerfua SD, Shirima G, Kusiluka L, Ayebazibwe C, Martin E, Arinaitwe E, Cleaveland S, Haydon DT (2019) Low topotype diversity of recent foot-and-mouth disease virus serotypes O and A from districts located along the Uganda and Tanzania border. J Vet Sci 20:e4 30944527 10.4142/jvs.2019.20.e4 PMC6441803

[81] Hassan AM, Zaher MR, Hassanien RT, Abd-El-Moniem MI, Habashi AR, Ibraheem EM, Shahein MA, El Zowalaty ME, Hagag NM (2022) Molecular detection, phylogenetic analysis and genetic diversity of recently isolated foot-and-mouth disease virus serotype A African topotype, genotype IV. J Virol 19:1 10.1186/s12985-021-01693-y PMC8722054 34980196

[82] Soobhy NM, Bayoumi YH, Mor SK, El-Zahar HI, Goyal SM (2018) Outbreaks of foot-and-mouth disease in Egypt: molecular epidemiology, evolution and cardiac biomarkers prognostic significance. Int J Vet Sci Med 6:22–30 30255074 10.1016/j.ijvsm.2018.02.001 PMC6148740

[83] El-Ansary RE, Kasem S, El-Tabakh MAM, Badr Y, Abdel-Moneim AS (2023) Isolation, molecular characterization, and genetic diversity of recently isolated foot-and-mouth disease virus serotype A in Egypt. PLoS One 18:e0295319 38051725 10.1371/journal.pone.0295319 PMC10697586

[84] Zhang X, Ma W, Yang F, Yang Y, Lv L, Wu J, Liu B, Shen C, Liu Y, Zhu Z, Shang Y, Guo J, Liu X, Zheng H, He J (2023) Epidemiological and genetic analysis of foot-and-mouth disease virus O/ME-SA/Ind-2001 in China between 2017 and 2021. Transbound Emerg Dis 2023:3761703 40303764 10.1155/2023/3761703 PMC12016701

[85] Eltahir YM, Ishag HZA, Wadsworth J, Hicks HM, Knowles NJ, Mioulet V, King DP, Mohamed MS, Bensalah OK, Yusof MF, Gasim EFM, Hammadi ZMA, Shah AAM, Abdelmagid YA, Gahlan MAME, Kassim MF, Kayaf K, Zahran A, Nuaimat MMA (2024) Molecular epidemiology of foot-and-mouth disease viruses in the emirate of Abu Dhabi, United Arab Emirates. Vet Sci 11:32 38250938 10.3390/vetsci11010032 PMC11154577

[86] Ali W, Habib M, Khan RSA, Zia MA, Farooq M, Sajid S, Shah MSUD (2018) Molecular investigation of foot-and-mouth disease virus circulating in Pakistan during 2014–17. Arch Virol 163:1733–1743 29516248 10.1007/s00705-018-3775-0

[87] Udahemuka JC, Aboge G, Obiero G, Ingabire A, Beeton N, Uwibambe E, Lebea P (2022) Investigation of foot-and-mouth disease virus and other animal pathogens in cattle, buffaloes and goats at the interface with Akagera National Park, 2017–2020. BMC Vet Res 18:349 36114497 10.1186/s12917-022-03430-1 PMC9479285

[88] Vu LT, Long NT, Brito B, Stenfeldt C, Phuong NT, Hoang BH, Pauszek SJ, Hartwig EJ, Smoliga GR, Vu PP, Quang LTV, Hung VV, Tho ND, Dong PV, Minh PQ, Bertram M, Fish IH, Rodriguez LL, Dung DH, Arzt J (2017) First detection of foot-and-mouth disease virus O/IND-2001D in Vietnam. PLoS One 12:e0177361 28599321 10.1371/journal.pone.0177361 PMC5466432

[89] Kimura M (1977) Preponderance of synonymous changes as evidence for the neutral theory of molecular evolution. Nature 267:275–276 865622 10.1038/267275a0

[90] Miyata T, Yasunaga T (1980) Molecular evolution of mRNA: a method for estimating evolutionary rates of synonymous and amino acid substitutions from homologous nucleotide sequences and its application. J Mol Evol 16:23–36 6449605 10.1007/BF01732067

[91] Subramaniam S, Mohapatra JK, Das B, Sharma GK, Biswal JK, Mahajan S, Misri J, Dash BB, Pattnaik B (2015) Capsid coding region diversity of re-emerging lineage C foot-and-mouth disease virus serotype Asia1 from India. Arch Virol 160:1751–1759 26008211 10.1007/s00705-015-2459-2

[92] Lycett S, Tanya V, Hall M, King D, Mazeri S, Mioulet V, Knowles N, Wadsworth J, Bachanek-Bankowska K, Ngu Ngwa V, Morgan K, Bronsvoort BMdC (2019) The evolution and phylodynamics of serotype A and SAT2 foot-and-mouth disease viruses in endemic regions of Africa. Sci Rep 9:5614 30948742 10.1038/s41598-019-41995-4 PMC6449503

[93] Das B, Mohapatra JK, Pande V, Subramaniam S, Sanyal A (2016) Evolution of foot-and-mouth disease virus serotype A capsid coding (P1) region on a timescale of three decades in an endemic context. Infect Genet Evol 41:36–46 27020544 10.1016/j.meegid.2016.03.024

[94] Biswal JK, Ranjan R, Subramaniam S, Mohapatra JK, Patidar S, Sharma MK, Bertram MR, Brito B, Rodriguez LL, Pattnaik B, Arzt J (2019) Genetic and antigenic variation of foot-and-mouth disease virus during persistent infection in naturally infected cattle and Asian buffalo in India. PLoS One 14:e0214832 31226113 10.1371/journal.pone.0214832 PMC6588224

[95] Brito B, Pauszek SJ, Eschbaumer M, Stenfeldt C, De Carvalho Ferreira HC, Vu LT, Phuong NT, Hoang BH, Tho ND, Dong PV, Minh PQ, Long NT, King DP, Knowles NJ, Dung DH, Rodriguez LL, Arzt J (2017) Phylodynamics of foot-and- mouth disease virus O/PanAsia in Vietnam 2010–2014. Vet Res 48:24 28403902 10.1186/s13567-017-0424-7 PMC5390394

[96] Pedersen CET, Frandsen P, Wekesa SN, Heller R, Sangula AK, Wadsworth J, Knowles NJ, Muwanika VB, Siegismund HR (2015) Time clustered sampling can inflate the inferred substitution rate in foot-and-mouth disease virus analyses. PLoS One 10:e0143605 26630483 10.1371/journal.pone.0143605 PMC4667911

[97] Lemey P, Rambaut A, Drummond AJ, Suchard MA (2009) Bayesian phylogeography finds its roots. PLoS Comput Biol 5:e1000520 19779555 10.1371/journal.pcbi.1000520 PMC2740835

[98] Bae S, Li V, Hong J, Kim JN, Kim H (2021) Phylogenetic and evolutionary analysis of foot-and-mouth disease virus A/ASIA/Sea-97 lineage. Virus Genes 57:443–447 34260046 10.1007/s11262-021-01848-7 PMC8445868

[99] Brito BP, Jori F, Dwarka R, Maree FF, Heath L, Perez AM (2016) Transmission of foot-and-mouth disease SAT2 viruses at the wildlife–livestock interface of two major transfrontier conservation areas in southern Africa. Front Microbiol 7:528 27148217 10.3389/fmicb.2016.00528 PMC4840674

[100] Jara M, Frias-De-Diego A, Dellicour S, Baele G, Machado G (2020) Tracing foot-and-mouth disease virus phylogeographical patterns and transmission dynamics. bioRxiv. 10.1101/590612

[101] Gunasekara U, Bertram MR, Van Long N, Minh PQ, Chuong VD, Perez A, Arzt J, VanderWaal K (2023) Phylogeography as a proxy for population connectivity for spatial modeling of foot-and-mouth disease outbreaks in Vietnam. Viruses 15:388 36851602 10.3390/v15020388 PMC9958845

[102] Lemey P, Rambaut A, Bedford T, Faria N, Bielejec F, Baele G, Russell CA, Smith DJ, Pybus OG, Brockmann D, Suchard MA (2014) Unifying viral genetics and human transportation data to predict the global transmission dynamics of human influenza H3N2. PLoS Pathog 10:e1003932 24586153 10.1371/journal.ppat.1003932 PMC3930559

[103] Duchatel F, Bronsvoort BMdC, Lycett S (2019) Phylogeographic analysis and identification of factors impacting the diffusion of foot-and-mouth disease virus in Africa. Front Ecol Evol 7:371

[104] Dellicour S, Rose R, Faria NR, Lemey P, Pybus OG (2016) SERAPHIM: studying environmental rasters and phylogenetically informed movements. Bioinformatics 32:3204–3206 27334476 10.1093/bioinformatics/btw384

[105] Gizaw D, Tesfaye Y, Wood BA, Di Nardo A, Shegu D, Muluneh A, Bilata T, Belayneh R, Fentie A, Asgdome H, Sombo M, Rufael T, Tadesse Woldemariyam F, Khan F, Yami M, Gelaye E, Wadsworth J, Knowles NJ, King DP (2020) Molecular characterization of foot-and-mouth disease viruses circulating in Ethiopia between 2008 and 2019. Transbound Emerg Dis 67:2983–2992 32574400 10.1111/tbed.13675

[106] Munsey A, Mwiine FN, Ochwo S, Velazquez-Salinas L, Ahmed Z, Maree F, Rodriguez LL, Rieder E, Perez A, Dellicour S, VanderWaal K (2021) Phylogeographic analysis of foot-and-mouth disease virus serotype O dispersal and associated drivers in East Africa. Mol Ecol 30:3815–3825 34008868 10.1111/mec.15991

[107] Munsey A, Mwiine FN, Ochwo S, Velazquez-Salinas L, Ahmed Z, Rodriguez LL, Rieder E, Perez A, VanderWaal K (2022) Ecological and anthropogenic spatial gradients shape patterns of dispersal of foot-and-mouth disease virus in Uganda. Pathogens 11:524 35631045 10.3390/pathogens11050524 PMC9143568

[108] Lau MSY, Marion G, Streftaris G, Gibson G (2015) A systematic Bayesian integration of epidemiological and genetic data. PLoS Comput Biol 11:e1004633 26599399 10.1371/journal.pcbi.1004633 PMC4658172

[109] De Maio N, Wu CH, Wilson DJ (2016) SCOTTI: efficient reconstruction of transmission within outbreaks with the structured coalescent. PLoS Comput Biol 12:e1005130 27681228 10.1371/journal.pcbi.1005130 PMC5040440

[110] Kenah E, Britton T, Halloran ME, Longini IM (2016) Molecular infectious disease epidemiology: survival analysis and algorithms linking phylogenies to transmission trees. PLoS Comput Biol 12:e1004869 27070316 10.1371/journal.pcbi.1004869 PMC4829193

[111] Klinkenberg D, Backer JA, Didelot X, Colijn C, Wallinga J (2017) Simultaneous inference of phylogenetic and transmission trees in infectious disease outbreaks. PLoS Comput Biol 13:e1005495 28545083 10.1371/journal.pcbi.1005495 PMC5436636

[112] Cottam EM, Thébaud G, Wadsworth J, Gloster J, Mansley L, Paton DJ, King DP, Haydon DT (2008) Integrating genetic and epidemiological data to determine transmission pathways of foot-and-mouth disease virus. Proc Biol Sci 275:887–895 18230598 10.1098/rspb.2007.1442 PMC2599933

[113] Cottam EM, Wadsworth J, Shaw AE, Rowlands RJ, Goatley L, Maan S, Maan NS, Mertens PPC, Ebert K, Li Y, Ryan ED, Juleff N, Ferris NP, Wilesmith JW, Haydon DT, King DP, Paton DJ, Knowles NJ (2008) Transmission pathways of foot-and-mouth disease virus in the United Kingdom in 2007. PLoS Pathog 4:e1000050 18421380 10.1371/journal.ppat.1000050 PMC2277462

[114] Valdazo-Gonzalez B, Polihronova L, Alexandrov T, Normann P, Knowles NJ, Hammond JM, Georgiev GK, Ozyoruk F, Sumption KJ, Belsham GJ, King DP (2012) Reconstruction of the transmission history of RNA virus outbreaks using full genome sequences: foot-and-mouth disease virus in Bulgaria in 2011. PLoS One 7:e49650 23226216 10.1371/journal.pone.0049650 PMC3511503

[115] Valdazo-Gonzalez B, Kim JT, Soubeyrand S, Wadsworth J, Knowles NJ, Haydon DT, King DP (2015) The impact of within-herd genetic variation upon inferred transmission trees for foot-and-mouth disease virus. Infect Genet Evol 32:440–448 25861750 10.1016/j.meegid.2015.03.032 PMC7106308

[116] Mateu MG, Camarero JA, Giralt E, Andreu D, Domingo E (1995) Direct evaluation of the immunodominance of a major antigenic site of foot-and-mouth disease virus in a natural host. Virology 206:298–306 7831785 10.1016/s0042-6822(95)80045-x

[117] Baxt B, Morgan DO, Robertson BH, Timpone CA (1984) Epitopes on foot-and-mouth disease virus outer capsid protein VP1 involved in neutralization and cell attachment. J Virol 51:298–305 6205165 10.1128/jvi.51.2.298-305.1984 PMC254438

[118] Knowles N, Samuel A (2003) Molecular epidemiology of foot-and-mouth disease virus. Virus Res 91:65–80 12527438 10.1016/s0168-1702(02)00260-5

[119] Stram Y, Molad T, Chai D, Gelman B, Yadin H (1995) Detection and subtyping of foot-and-mouth disease virus in infected cattle by polymerase chain reaction and amplified VP1 sequencing. J Vet Diagn Invest 7:52–55 7779964 10.1177/104063879500700107

[120] Marquardt O, Adam KH (1990) Foot-and-mouth disease virus subtyping by sequencing vp1 genes. Vet Microbiol 23:175–183 2169671 10.1016/0378-1135(90)90147-n

[121] Peng J, Yi J, Yang W, Ren J, Wen Y, Zheng H, Li D (2020) Advances in foot-and-mouth disease virus proteins regulating host innate immunity. Front Microbiol 11:2046 33162944 10.3389/fmicb.2020.02046 PMC7581685

[122] Liu Y, Zhu Z, Zhang M, Zheng H (2015) Multifunctional roles of leader protein of foot-and-mouth disease viruses in suppressing host antiviral responses. Vet Res 46:127 26511922 10.1186/s13567-015-0273-1 PMC4625562

[123] Gao Y, Sun SQ, Guo HC (2016) Biological function of foot-and-mouth disease virus non-structural proteins and non-coding elements. J Virol 13:107 10.1186/s12985-016-0561-z PMC4917953 27334704

[124] Kloc A, Diaz-San Segundo F, Schafer EA, Rai DK, Kenney M, de los Santos T, Rieder E (2017) Foot-and-mouth disease virus 5’-terminal s fragment is required for replication and modulation of the innate immune response in host cells. Virology 512:132–143 28961454 10.1016/j.virol.2017.08.036

[125] Posada D, Crandall KA (2002) The effect of recombination on the accuracy of phylogeny estimation. J Mol Evol 54:396–402 11847565 10.1007/s00239-001-0034-9

[126] Schierup MH, Hein J (2000) Consequences of recombination on traditional phylogenetic analysis. Genetics 156:879–891 11014833 10.1093/genetics/156.2.879 PMC1461297

[127] Freimanis G, Di Nardo A, Bankowska K, King D, Wadsworth J, Knowles N, King D (2016) Genomics and outbreaks: foot-and-mouth disease. Rev Sci Tech 35:175–189 27217177 10.20506/rst.35.1.2426

[128] Brito B, Pauszek SJ, Hartwig EJ, Smoliga GR, Vu LT, Dong PV, Stenfeldt C, Rodriguez LL, King DP, Knowles NJ, Bachanek-Bankowska K, Long NT, Dung DH, Arzt J (2018) A traditional evolutionary history of foot-and-mouth disease viruses in Southeast Asia challenged by analyses of non-structural protein coding sequences. Sci Rep 8:6472 29691483 10.1038/s41598-018-24870-6 PMC5915611

[129] Wang Z, Liu KJ (2016) A performance study of the impact of recombination on species tree analysis. BMC Genomics 17:785 28185556 10.1186/s12864-016-3104-5 PMC5123380

[130] Heath L, Van Der Walt E, Varsani A, Martin DP (2006) Recombination patterns in aphthoviruses mirror those found in other picornaviruses. J Virol 80:11827–11832 16971423 10.1128/JVI.01100-06 PMC1642601

[131] Aiewsakun P, Pamornchainavakul N, Inchaisri C (2020) Early origin and global colonisation of foot-and-mouth disease virus. Sci Rep 10:15268 32943727 10.1038/s41598-020-72246-6 PMC7498456

[132] Lasecka-Dykes L, Wright CF, Di Nardo A, Logan G, Mioulet V, Jackson T, Tuthill TJ, Knowles NJ, King DP (2018) Full genome sequencing reveals new southern African territories genotypes bringing us closer to understanding true variability of foot-and-mouth disease virus in Africa. Viruses 10:192 29652800 10.3390/v10040192 PMC5923486

[133] Delahaye C, Nicolas J (2021) Sequencing DNA with nanopores: troubles and biases. PLoS One 16:e0257521 34597327 10.1371/journal.pone.0257521 PMC8486125

[134] Chin CS, Alexander DH, Marks P, Klammer AA, Drake J, Heiner C, Clum A, Copeland A, Huddleston J, Eichler EE, Turner SW, Korlach J (2013) Nonhybrid, finished microbial genome assemblies from long-read SMRT sequencing data. Nat Methods 10:563–569 23644548 10.1038/nmeth.2474

[135] Woldemariyam FT, Kariuki CK, Kamau J, De Vleeschauwer A, De Clercq K, Lefebvre DJ, Paeshuyse J (2023) Epidemiological dynamics of foot-and-mouth disease in the horn of Africa: the role of virus diversity and animal movement. Viruses 15:969 37112947 10.3390/v15040969 PMC10143177

[136] Omondi GP, Gakuya F, Arzt J, Sangula A, Hartwig E, Pauszek S, Smoliga G, Brito B, Perez A, Obanda V, VanderWaal K (2020) The role of African buffalo in the epidemiology of foot-and-mouth disease in sympatric cattle and buffalo populations in Kenya. Transbound Emerg Dis 67:2206–2221 32303117 10.1111/tbed.13573

[137] Naqvi SS, Bostan N, Fukai K, Ali Q, Morioka K, Nishi T, Abubakar M, Ahmed Z, Sattar S, Javed S (2022) Evolutionary dynamics of foot-and-mouth disease virus serotype A and its endemic sub-lineage A/ASIA/Iran-05/SIS-13 in Pakistan. Viruses 14:1634 35893699 10.3390/v14081634 PMC9331208

[138] Alexandrov T, Stefanov D, Kamenov P, Miteva A, Khomenko S, Sumption K, Meyer-Gerbaulet H, Depner K (2013) Surveillance of foot-and-mouth disease (FMD) in susceptible wildlife and domestic ungulates in Southeast of Bulgaria following a FMD case in wild boar. Vet Microbiol 166:84–90 23830685 10.1016/j.vetmic.2013.05.016

[139] Clemmons EA, Alfson KJ, Dutton JW (2021) Transboundary animal diseases, an overview of 17 diseases with potential for global spread and serious consequences. Animals 11:2039 34359167 10.3390/ani11072039 PMC8300273

[140] van Dam A, van Engelen W, Muller-Mahn D, Agha S, Junglen S, Borgemeister C, Bollig M (2024) Complexities of multispecies coexistence: animal diseases and diverging modes of ordering at the wildlife–livestock interface in Southern Africa. Environ Plan E-Nat 7:353–374

[141] Knight-Jones TJD, Robinson L, Charleston B, Rodriguez LL, Gay CG, Sumption KJ, Vosloo W (2016) Global foot-and-mouth disease research update and gap analysis: 2 - epidemiology, wildlife and economics. Transbound Emerg Dis 63:14–29 27320163 10.1111/tbed.12522

[142] Atuman YJ, Kudi CA, Abdu PA, Okubanjo OO, Abubakar A, Wungak Y, Ularamu HG (2020) Seroprevalence of foot and mouth disease virus infection in some wildlife and cattle in Bauchi State, Nigeria. Vet Med Int 2020:3642793 32257095 10.1155/2020/3642793 PMC7104331

[143] Rahman Au, Dhama K, Ali Q, Raza MA, Chaudhry U, Shabbir MZ (2020) Foot-and-mouth disease in a wide range of wild hosts: a potential constraint in disease control efforts worldwide, particularly in disease-endemic settings. Acta Trop 210:105567 32504589 10.1016/j.actatropica.2020.105567

[144] Brown VR, Miller RS, McKee SC, Ernst KH, Didero NM, Maison RM, Grady MJ, Shwiff SA (2021) Risks of introduction and economic consequences associated with African swine fever, classical swine fever and foot-and-mouth disease: a review of the literature. Transbound Emerg Dis 68:1910–1965 33176063 10.1111/tbed.13919

[145] Thomson GR, Vosloo W, Bastos ADS (2003) Foot-and-mouth disease in wildlife. Virus Res 91:145–161 12527441 10.1016/s0168-1702(02)00263-0

[146] Jori F, Hernandez-Jover M, Magouras I, Dürr S, Brookes VJ (2021) Wildlife–livestock interactions in animal production systems: what are the biosecurity and health implications? Anim Front 11:8–19 34676135 10.1093/af/vfab045 PMC8527523

[147] Duchatel F, Maree F, Schalkwyk LV, Bronsvoort BM, Lycett S (2023) Importance of wildlife in the circulation and maintenance of SAT1 and SAT2 foot-and-mouth disease viruses in Africa. biorxiv. 10.1101/2023.05.01.538841

[148] Mohamed F, Swafford S, Petrowski H, Bracht A, Schmit B, Fabian A, Pacheco JM, Hartwig E, Berninger M, Carrillo C, Mayr G, Moran K, Kavanaugh D, Leibrecht H, White W, Metwally S (2011) Foot-and-mouth disease in feral swine: susceptibility and transmission. Transbound Emerg Dis 58:358–371 21418546 10.1111/j.1865-1682.2011.01213.x

[149] Fukai K, Kawaguchi R, Nishi T, Ikezawa M, Yamada M, Seeyo KB, Morioka K (2022) Risk of transmission of foot-and-mouth disease by wild animals: infection dynamics in Japanese wild boar following direct inoculation or contact exposure. Vet Res 53:86 36273214 10.1186/s13567-022-01106-0 PMC9587633

[150] Cortazar C, Barroso P, Nova R, Cáceres G (2022) The role of wildlife in the epidemiology and control of Foot-and-mouth-disease And Similar Transboundary (FAST) animal diseases: a review. Transbound Emerg Dis 69:2462–2473 34268873 10.1111/tbed.14235

[151] Karniely S, Hamed F, Gelman B, King R, Storm N, Eyngor E, Even Tov B (2020) First isolation of foot-and-mouth disease virus from wild boars in the Middle East. Transbound Emerg Dis 67:1725–1729 32034998 10.1111/tbed.13507

[152] Miller RS, Bevins SN, Cook G, Free R, Pepin KM, Gidlewski T, Brown VR (2022) Adaptive risk-based targeted surveillance for foreign animal diseases at the wildlife-livestock interface. Transbound Emerg Dis 69:e2329–e2340 35490290 10.1111/tbed.14576 PMC9790623

[153] Rhyan J, McCollum M, Gidlewski T, Shalev M, Ward G, Donahue B, Arzt J, Stenfeldt C, Mohamed F, Nol P, Deng M, Metwally S, Salman M (2019) Foot-and-mouth disease in experimentally infected mule deer (*Odocoileus hemionus*). J Wildl Dis 56:93–104 31329525

[154] Highfield LD, Ward MP, Laffan SW, Norby B, Wagner G (2009) The impact of seasonal variability in wildlife populations on the predicted spread of foot-and-mouth disease. Vet Res 40:18 19134466 10.1051/vetres:2009001 PMC2695039

[155] Rout M, Karikalan M, Manjunatha V, Sahoo N, Nair N, Mohapatra J, Dash B, Sharma A, Singh R (2024) Serological profiling of foot and mouth disease virus nonstructural protein antibodies in susceptible wild or captive ruminants in India. Indian J Vet Pathol 48:176–180

[156] Triguero-Ocana R, Vicente J, Lavelle M, Acevedo P (2021) Collecting data to assess the interactions between livestock and wildlife. In: Vicente J, Vercauteren KC, Gortazar C (eds) Diseases at the wildlife-livestock interface: research and perspectives in a changing world. Springer International Publishing, Cham, pp 307–338

[157] Mashinagu MM, Wambura PN, King DP, Paton DJ, Maree F, Kimera SI, Rweyemamu MM, Kasanga CJ (2024) Challenges of controlling foot-and-mouth disease in pastoral settings in Africa. Transbound Emerg Dis 2024:2700985 40303029 10.1155/2024/2700985 PMC12017246

[158] Khanyari M, Robinson S, Morgan ER, Brown T, Singh NJ, Salemgareyev A, Zuther S, Kock R, Milner-Gulland EJ (2021) Building an ecologically founded disease risk prioritization framework for migratory wildlife species based on contact with livestock. J Appl Ecol 58:1838–1853

[159] Waters RA, Wadsworth J, Mioulet V, Shaw AE, Knowles NJ, Abdollahi D, Has-Sanzadeh R, Sumption K, King DP (2021) Foot-and-mouth disease virus infection in the domestic dog (*Canis lupus familiaris*), Iran. BMC Vet Res 17:63 33526020 10.1186/s12917-021-02769-1 PMC7852191

[160] Aslam M, Alkheraije KA (2023) The prevalence of foot-and-mouth disease in Asia. Front Vet Sci 10:1201578 37456961 10.3389/fvets.2023.1201578 PMC10347409

[161] Manyenya S, Nthiwa D, Lutta HO, Muturi M, Nyamota R, Mwatondo A, Watene G, Akoko J, Bett B (2024) Multiple pathogens co-exposure and associated risk factors among cattle reared in a wildlife-livestock interface area in Kenya. Front Vet Sci 11:1415423 39119353 10.3389/fvets.2024.1415423 PMC11306132

[162] Haoran W, Jianhua X, Maolin O, Hongyan G, Jia B, Li G, Xiang G, Hongbin W (2021) Assessment of foot-and-mouth disease risk areas in mainland China based spatial multi-criteria decision analysis. BMC Vet Res 17:374 34872574 10.1186/s12917-021-03084-5 PMC8647368

[163] Gonzalez Gordon L, Porphyre T, Muhanguzi D, Muwonge A, Boden L, Bronsvoort BMDC (2022) A scoping review of foot-and-mouth disease risk, based on spatial and spatio-temporal analysis of outbreaks in endemic settings. Transbound Emerg Dis 69:3198–3215 36383164 10.1111/tbed.14769 PMC10107783

[164] Hayes BH, Vergne T, Andraud M, Rose N (2023) Mathematical modeling at the livestock-wildlife interface: scoping review of drivers of disease transmission between species. Front Vet Sci 10:1225446 37745209 10.3389/fvets.2023.1225446 PMC10511766

[165] Zaheer MU, Salman MD, Steneroden KK, Magzamen SL, Weber SE, Case S, Rao S (2020) Challenges to the application of spatially explicit stochastic simulation models for foot-and-mouth disease control in endemic settings: a systematic review. Comput Math Methods Med 2020:7841941 33294003 10.1155/2020/7841941 PMC7700052

[166] van Andel M, Tildesley MJ, Gates MC (2021) Challenges and opportunities for using national animal datasets to support foot-and-mouth disease control. Transbound Emerg Dis 68:1800–1813 32986919 10.1111/tbed.13858

[167] Hoogesteyn AL, Rivas AL, Smith SD, Fasina FO, Fair JM, Kosoy M (2023) Assessing complexity and dynamics in epidemics: geographical barriers and facilitators of foot-and-mouth disease dissemination. Front Vet Sci 10:1149460 37252396 10.3389/fvets.2023.1149460 PMC10213354

[168] Sansamur C, Wiratsudakul A, Charoenpanyanet A, Punyapornwithaya V (2021) Estimating the number of farms experienced foot-and-mouth disease outbreaks using capture-recapture methods. Trop Anim Health Prod 53:12 10.1007/s11250-020-02452-x 33211202

[169] Meurens F, Dunoyer C, Fourichon C, Gerdts V, Haddad N, Kortekaas J, Lewandowska M, Monchatre-Leroy E, Summerfield A, Wichgers Schreur PJ, van der Poel WHM, Zhu J (2021) Animal board invited review: risks of zoonotic disease emergence at the interface of wildlife and livestock systems. Animal 15:100241 34091225 10.1016/j.animal.2021.100241 PMC8172357

[170] Yang A, Boughton RK, Miller RS, Wight B, Anderson WM, Beasley JC, VerCauteren KC, Pepin KM, Wittemyer G (2021) Spatial variation in direct and indirect contact rates at the wildlife-livestock interface for informing disease management. Prev Vet Med 194:105423 34246115 10.1016/j.prevetmed.2021.105423

[171] Compston P, Limon G, Sangula A, Onono J, King DP, Hasler B (2021) Understanding what shapes disease control: an historical analysis of foot-and-mouth disease in Kenya. Prev Vet Med 190:105315 33735817 10.1016/j.prevetmed.2021.105315

[172] Casey MB, Lembo T, Knowles NJ, Fyumagwa R, Kivaria F, Maliti H, Kasanga C, Sallu R, Reeve R, Parida S, King DP, Cleaveland S (2014) Patterns of foot-and-mouth disease virus distribution in Africa: the role of livestock and wildlife in virus emergence. In: Johnson N (ed) The role of animals in emerging viral diseases. Academic Press, Boston, pp 21–38

[173] Smyser TJ, Pfaffelhuber P, Giglio RM, DeSaix MG, Davis AJ, Bowden CF, Tabak MA, Manunza A, Balteanu VA, Amills M, Iacolina L, Walker P, Lessard C, Piaggio AJ (2024) Probabilistic genetic identification of wild boar hybridization to support control of invasive wild pigs (s*us scrofa)*. Ecosphere 15:e4774

[174] Giglio RM, Bowden CF, Brook RK, Piaggio AJ, Smyser TJ (2024) Characterizing feral swine movement across the contiguous United States using neural networks and genetic data. Mol Ecol 33:e17489 39148259 10.1111/mec.17489

[175] Brown VR, Marlow MC, Gidlewski T, Bowen R, Bosco-Lauth A (2020) Perspectives on the past, present, and future of feral swine disease surveillance in the United States. J Anim Sci 98:skaa256 32857859 10.1093/jas/skaa256 PMC7454954

[176] Jiang F, Song P, Zhang J, Cai Z, Chi X, Gao H, Qin W, Li S, Zhang T (2020) Assessing the impact of climate change on the spatio-temporal distribution of foot-and-mouth disease risk for elephants. Glob Ecol Conserv 23:e01176

[177] Bates TW, Thurmond MC, Carpenter TE (2003) Results of epidemic simulation modeling to evaluate strategies to control an outbreak of foot-and-mouth disease. Am J Vet Res 64:205–210 12602590 10.2460/ajvr.2003.64.205

[178] Garner M, Beckett S (2005) Modelling the spread of foot-and-mouth disease in Australia. Aust Vet J 83:758–766 16395942 10.1111/j.1751-0813.2005.tb11589.x

[179] Harvey N, Reeves A, Schoenbaum MA, Zagmutt-Vergara FJ, Dube C, Hill AE, Corso BA, McNab WB, Cartwright CI, Salman MD (2007) The North American Animal Disease Spread Model: a simulation model to assist decision making in evaluating animal disease incursions. Prev Vet Med 82:176–197 17614148 10.1016/j.prevetmed.2007.05.019

[180] Tildesley MJ, Deardon R, Savill NJ, Bessell PR, Brooks SP, Woolhouse ME, Grenfell BT, Keeling MJ (2008) Accuracy of models for the 2001 foot-and-mouth epidemic. Proc Biol Sci 275:1459–1468 18364313 10.1098/rspb.2008.0006 PMC2376304

[181] Stevenson M, Sanson R, Stern M, O’Leary B, Sujau M, Moles-Benfell N, Morris R (2013) InterSpread Plus: a spatial and stochastic simulation model of disease in animal populations. Prev Vet Med 109:10–24 22995473 10.1016/j.prevetmed.2012.08.015

[182] Buhnerkempe MG, Tildesley MJ, Lindström T, Grear DA, Portacci K, Miller RS, Lombard JE, Werkman M, Keeling MJ, Wennergren U, Webb CT (2014) The impact of movements and animal density on continental scale cattle disease outbreaks in the United States. PLoS One 9:e91724 24670977 10.1371/journal.pone.0091724 PMC3966763

[183] Bradhurst RA, Roche SE, East IJ, Kwan P, Garner MG (2015) A hybrid modeling approach to simulating foot-and-mouth disease outbreaks in Australian livestock. Front Environ Sci 3:17

[184] Bradhurst R, Garner G, Hovari M, De La Puente M, Mintiens K, Yadav S, Federici T, Kopacka I, Stockreiter S, Kuzmanova I, Paunov S, Cacinovic V, Rubin M, Szilagyi J, Kokany ZS, Santi A, Sordilli M, Sighinas L, Spiridon M, Potocnik M, Sumption K (2022) Development of a transboundary model of livestock disease in Europe. Transbound Emerg Dis 69:1963–1982 34169659 10.1111/tbed.14201 PMC9545780

[185] Conrady B, Mortensen S, Nielsen SS, Houe H, Calvo-Artavia FF, Ellis-Iversen J, Boklund A (2023) Simulation of foot-and-mouth disease spread and effects of mitigation strategies to support veterinary contingency planning in Denmark. Pathogens 12:435 36986357 10.3390/pathogens12030435 PMC10056164

[186] Tsao K, Sellman S, Beck-Johnson LM, Murrieta DJ, Hallman C, Lindström T, Miller RS, Portacci K, Tildesley MJ, Webb CT (2020) Effects of regional differences and demography in modelling foot-and-mouth disease in cattle at the national scale. Interface Focus 10:20190054 31897292 10.1098/rsfs.2019.0054 PMC6936011

[187] Seibel RL, Meadows AJ, Mundt C, Tildesley M (2024) Modeling target- density-based cull strategies to contain foot-and-mouth disease outbreaks. PeerJ 12:e16998 38436010 10.7717/peerj.16998 PMC10909358

[188] Hafi A, Addai D, Breed A, Bradhurst R, Capon T, Garner M, Miller C, Pinol J, Seitzinger A, Tapsuwan S (2022) Economic benefits of implementing trading zones for Australian livestock disease outbreaks of limited duration. Aust Vet J 100:150–161 35049045 10.1111/avj.13141 PMC9303469

[189] Sanson RL, Rawdon TG, van Andel M, Yu Z (2022) Modelling the field personnel resources to control foot-and-mouth disease outbreaks in New Zealand. Transbound Emerg Dis 69:3926–3939 36397293 10.1111/tbed.14764

[190] Beck-Johnson LM, Gorsich EE, Hallman C, Tildesley MJ, Miller RS, Webb CT (2023) An exploration of within-herd dynamics of a transboundary livestock disease: a foot-and-mouth disease case study. Epidemics 42:100668 36696830 10.1016/j.epidem.2023.100668

[191] Walz E, Evanson J, Sampedro F, VanderWaal K, Goldsmith T (2020) Planning “Plan B”: the case of moving cattle from an infected feedlot premises during a hypothetical widespread FMD outbreak in the United States. Front Vet Sci 6:484 31998764 10.3389/fvets.2019.00484 PMC6964524

[192] Cabezas AH, Sanderson MW, Volkova VV (2020) A meta-population model of potential foot-and-mouth disease transmission, clinical manifestation, and detection within U.S. beef feedlots. Front Vet Sci 7:527558 33195510 10.3389/fvets.2020.527558 PMC7543087

[193] Cabezas AH, Sanderson MW, Volkova VV (2021) Modeling intervention scenarios during potential foot-and-mouth disease outbreaks within U.S. beef feedlots. Front Vet Sci 8:559785 33665214 10.3389/fvets.2021.559785 PMC7921729

[194] Mielke SR, Rigney C, Hagerman AD, Boyer TC, Delgado AH, Arzt J, Holmstrom LK (2023) Assessment of a reconfiguration of the InterSpread Plus US national FMD model as a potential tool to analyze a foot-and-mouth disease outbreak on a single large cattle feedlot in the United States. Front Vet Sci 10:1205485 37662981 10.3389/fvets.2023.1205485 PMC10468568

[195] Hagerman AD, South DD, Sondgerath TC, Patyk KA, Sanson RL, Schumacher RS, Delgado AH, Magzamen S (2018) Temporal and geographic distribution of weather conditions favorable to airborne spread of foot-and-mouth disease in the coterminous United States. Prev Vet Med 161:41–49 30466657 10.1016/j.prevetmed.2018.10.016

[196] Coffman M, Sanderson M, Dodd C, Arzt J, Renter D (2021) Estimation of foot-and-mouth disease windborne transmission risk from USA beef feedlots. Prev Vet Med 195:105453 34479032 10.1016/j.prevetmed.2021.105453

[197] Chanchaidechachai T, Saatkamp HW, Hogeveen H, de Jong MC, Fischer EA (2023) Evaluation of foot-and-mouth disease control measures: simulating two endemic areas of Thailand. Prev Vet Med 220:106045 37866130 10.1016/j.prevetmed.2023.106045

[198] Wongnak P, Yano T, Sekiguchi S, Chalvet-Monfray K, Premashthira S, Thanapongtharm W, Wiratsudakul A (2024) A stochastic modelling study of quarantine strategies against foot-and-mouth disease risks through cattle trades across the Thailand–Myanmar border. Prev Vet Med 230:106282 39033658 10.1016/j.prevetmed.2024.106282

[199] Gunasekera U, Biswal JK, Machado G, Ranjan R, Subramaniam S, Rout M, Mohapatra JK, Pattnaik B, Singh RP, Arzt J, Perez A, VanderWaal K (2022) Impact of mass vaccination on the spatiotemporal dynamics of FMD outbreaks in India, 2008–2016. Transbound Emerg Dis 69:e1936–e1950 35306749 10.1111/tbed.14528 PMC9790522

[200] Pomeroy LW, Kim H, Xiao N, Moritz M, Garabed R (2019) Network analyses to quantify effects of host movement in multilevel disease transmission models using foot-and-mouth disease in Cameroon as a case study. PLoS Comput Biol 15:e1007184 31465448 10.1371/journal.pcbi.1007184 PMC6776348

[201] Pomeroy LW, Moritz M, Garabed R (2019) Network analyses of transhumance movements and simulations of foot-and-mouth disease virus transmission among mobile livestock in Cameroon. Epidemics 28:100334 31387783 10.1016/j.epidem.2019.02.005

[202] Do H, Nguyen HTM, Van Ha P, Kompas T, Van KD, Chu L (2022) Estimating the transmission parameters of foot-and-mouth disease in Vietnam: a spatial-dynamic kernel-based model with outbreak and host data. Prev Vet Med 208:105773 36228512 10.1016/j.prevetmed.2022.105773

[203] Sangrat W, Thanapongtharm W, Poolkhet C (2020) Identification of risk areas for foot-and-mouth disease in Thailand using a geographic information system-based multi-criteria decision analysis. Prev Vet Med 185:105183 33153767 10.1016/j.prevetmed.2020.105183

[204] Punyapornwithaya V, Klaharn K, Arjkumpa O, Sansamur C (2022) Exploring the predictive capability of machine learning models in identifying foot-and-mouth disease outbreak occurrences in cattle farms in an endemic setting of Thailand. Prev Vet Med 207:105706 35863259 10.1016/j.prevetmed.2022.105706

[205] van Andel M, Zaari S, Bernard P, McFadden A, Dacre I, Bingham P, Heuer C, Binney B, Buckle K, Abila R, Win HH, Lwin KO, Gates MC (2020) Evaluating the utility of national-scale data to estimate the local risk of foot-and-mouth disease in endemic regions. Transbound Emerg Dis 67:108–120 31408585 10.1111/tbed.13329

[206] Brusa V, Durrieu M, Van Gelderen C, Signorini M, Schudel A (2023) Quantitative risk assessment of FMDV introduction in a FMD-free country through bone-in beef and offal importation from a FMD-free with vaccination country/zone. Prev Vet Med 218:105995 37625212 10.1016/j.prevetmed.2023.105995

[207] McKee SC, Brown VR, Shwiff SA, Giallombardo GM, Miller RS (2023) Areas within the United States at the highest risk for African swine fever, classical swine fever, and foot-and-mouth disease introduction. Transbound Emerg Dis 2023:8892037 40303669 10.1155/2023/8892037 PMC12016723

[208] Meyer A, Zamir L, Ben Yair Gilboa A, Gelman B, Pfeiffer DU, Vergne T (2017) Quantitative assessment of the risk of release of foot-and-mouth disease virus via export of bull semen from Israel. Risk Anal 37:2350–2359 28334452 10.1111/risa.12799

[209] Meyer A, Weiker J, Meyer R (2023) Laboratory testing and on-site storage are successful at mitigating the risk of release of foot-and-mouth disease virus via production of bull semen in the USA. PLoS One 18:e0294036 37934775 10.1371/journal.pone.0294036 PMC10629637

[210] ElAshmawy WR, Aly SS, Farouk MM (2023) Decision tree risk analysis for FMD outbreak prevention in Egyptian feedlots. Prev Vet Med 211:105820 36584568 10.1016/j.prevetmed.2022.105820

[211] Souley Kouato B, De Clercq K, Abatih E, Dal Pozzo F, King DP, Thys E, Marichatou H, Saegerman C (2018) Review of epidemiological risk models for foot-and-mouth disease: implications for prevention strategies with a focus on Africa. PLoS One 13:e0208296 30543641 10.1371/journal.pone.0208296 PMC6292601

[212] Kinsley AC, Patterson G, VanderWaal KL, Craft ME, Perez AM (2016) Parameter values for epidemiological models of foot-and-mouth disease in swine. Front Vet Sci 3:44 27314002 10.3389/fvets.2016.00044 PMC4887472

[213] Moreno-Torres KI, Brito BP, Branan MA, Rodriguez LL, Delgado AH, Stenfeldt C, Arzt J (2018) Foot-and-mouth disease infection dynamics in contact- exposed pigs are determined by the estimated exposure dose. Front Vet Sci 5:167 30079340 10.3389/fvets.2018.00167 PMC6062637

[214] Moreno-Torres KI, Delgado AH, Branan MA, Yadav S, Stenfeldt C, Arzt J (2022) Parameterization of the durations of phases of foot-and-mouth disease in pigs. Prev Vet Med 202:105615 35339769 10.1016/j.prevetmed.2022.105615

[215] Yadav S, Stenfeldt C, Branan MA, Moreno-Torres KI, Holmstrom LK, Delgado AH, Arzt J (2019) Parameterization of the durations of phases of foot-and- mouth disease in cattle. Front Vet Sci 6:263 31448297 10.3389/fvets.2019.00263 PMC6696987

[216] Cardenas NC, Valencio A, Sanchez F, O’Hara KC, Machado G (2024) Analyzing the intrastate and interstate swine movement network in the United States. Prev Vet Med 230:106264 39003835 10.1016/j.prevetmed.2024.106264

[217] Makau DN, Paploski IA, VanderWaal K (2021) Temporal stability of swine movement networks in the U.S. Prev Vet Med 191:105369 33965745 10.1016/j.prevetmed.2021.105369

[218] Kinsley A, Perez A, Craft M, Vanderwaal K (2019) Characterization of swine movements in the United States and implications for disease control. Prev Vet Med 164:1–9 30771888 10.1016/j.prevetmed.2019.01.001

[219] Iriarte MV, Gonzales JL, Gil AD, De Jong MCM (2023) Animal movements and FMDV transmission during the high-risk period of the 2001 FMD epidemic in Uruguay. Transbound Emerg Dis 2023:8883502 40303692 10.1155/2023/8883502 PMC12016683

[220] Wiltshire S, Zia A, Koliba C, Bucini G, Clark E, Merrill S, Smith J, Moegenburg S (2019) Network meta-metrics: using evolutionary computation to identify effective indicators of epidemiological vulnerability in a livestock production system model. J Artif Soc 22:8

[221] Valdes-Donoso P, VanderWaal K, Jarvis LS, Wayne SR, Perez AM (2017) Using machine learning to predict swine movements within a regional program to improve control of infectious diseases in the US. Front Vet Sci 4:2 28154817 10.3389/fvets.2017.00002 PMC5243845

[222] Gilbertson K, Brommesson P, Minter A, Hallman C, Miller RS, Portacci K, Sellman S, Tildesley MJ, Webb CT, Lindstrom T, Beck-Johnson LM (2022) The importance of livestock demography and infrastructure in driving foot and mouth disease dynamics. Life 12:1604 36295038 10.3390/life12101604 PMC9605081

[223] Kinsley AC, VanderWaal K, Craft ME, Morrison RB, Perez AM (2018) Managing complexity: simplifying assumptions of foot-and-mouth disease models for swine. Transbound Emerg Dis 65:1307–1317 29687629 10.1111/tbed.12880

[224] Meadows AJ, Mundt CC, Keeling MJ, Tildesley MJ (2018) Disentangling the influence of livestock vs. farm density on livestock disease epidemics. Ecosphere 9:e02294

[225] Sellman S, Tildesley MJ, Burdett CL, Miller RS, Hallman C, Webb CT, Wennergren U, Portacci K, Lindstrom T (2020) Realistic assumptions about spatial locations and clustering of premises matter for models of foot-and-mouth disease spread in the United States. PLoS Comput Biol 16:e1007641 32078622 10.1371/journal.pcbi.1007641 PMC7053778

[226] Han J, Subharat S, Wada M, Vink D, Phiri BJ, Sutar A, Abila R, Khounsy S, Heuer C (2022) Impact of risk-based partial vaccination on clinical incidence and seroprevalence of foot-and-mouth disease in Lao PDR. Transbound Emerg Dis 69:e309–e321 34412164 10.1111/tbed.14299

[227] Knight-Jones T, Rushton J (2013) The economic impacts of foot-and-mouth disease—what are they, how big are they and where do they occur? Prev Vet Med 112:161–173 23958457 10.1016/j.prevetmed.2013.07.013 PMC3989032

[228] National Audit Office (2002) The 2001 outbreak of foot-and-mouth disease. House of Commons Paper HC 939, Session 2001–2002

[229] Caro D, Davis SJ, Bastianoni S, Caldeira K (2014) Global and regional trends in greenhouse gas emissions from livestock. Clim Change 126:203–216

[230] Chanchaidechachai T, Fischer EA, Saatkamp HW, de Jong MC, Hogeveen H (2024) One-size measures do not fit all areas: evaluation of area-specific control of foot-and-mouth disease in Thailand using bioeconomic modelling. Prev Vet Med 233:106359 39437627 10.1016/j.prevetmed.2024.106359

[231] Rasmussen P, Shaw A, Jemberu W, Knight-Jones T, Conrady B, Apenteng O, Cheng Y, Muñoz V, Rushton J, Torgerson P (2024) Economic losses due to foot-and-mouth disease (FMD) in Ethiopian cattle. Prev Vet Med 230:106276 38991426 10.1016/j.prevetmed.2024.106276

[232] Compston P, Limon G, Hasler B (2022) A systematic review of the methods used to analyze the economic impact of endemic foot-and-mouth disease. Transbound Emerg Dis 69:e2249–e2260 35445543 10.1111/tbed.14564 PMC9795869

[233] Institute for Health Metrics and Evaluation (IHME) (2024) Global burden of disease 2021: findings from the GBD 2021 study. IHME

[234] Gilbert W, Marsh TL, Chaters G, Jemberu WT, Bruce M, Steeneveld W, Afonso JS, Huntington B, Rushton J (2024) Quantifying cost of disease in livestock: a new metric for the Global Burden of Animal Diseases. Lancet Planet Health 8:e309–e317 38729670 10.1016/S2542-5196(24)00047-0 PMC11636736

[235] Jemberu W, Chaters G, Asfaw W, Asteraye G, Amenu K, Huntington B, Knight-Jones T, Rushton J (2024) Application of global burden of animal diseases methods at country level: experiences of the Ethiopia case study. Rev Sci Tech 43:115–125 39222105 10.20506/rst.43.3524

[236] Aho PW (2002) The world’s commercial chicken meat and egg industries. Springer, Boston, pp 3–17

[237] Bruce M, Jemberu W, Larkins A (2024) A methodological framework for attributing the burden of animal disease to specific causes. Rev Sci Tech 43:48–57 39222112 10.20506/rst.43.3517

